# A Review on Environmental Occurrence and Toxicity of Perfluorooctanoic Acid and Its Selected Short-Chain Analogs—Perfluorohexanoic Acid and Perfluorobutanoic Acid

**DOI:** 10.3390/ijms27146221

**Published:** 2026-07-12

**Authors:** Izabela Kaczmarek, Katarzyna Mokra, Jaromir Michałowicz

**Affiliations:** 1 Department of Biophysics of Environmental Pollution, Faculty of Biology and Environmental Protection, University of Lodz, 141/143 Pomorska St., 90-236 Lodz, Poland; izabela.kaczmarek2@edu.uni.lodz.pl (I.K.); katarzyna.mokra@biol.uni.lodz.pl (K.M.); 2 Doctoral School of Exact and Natural Sciences, University of Lodz, Matejki 21/23 St., 90-237 Lodz, Poland

**Keywords:** perfluorooctanoic acid, perfluorohexanoic acid, perfluorobutanoic acid, environment, hepatotoxicity, epigenotoxicity, immunotoxicity

## Abstract

In this review, the occurrence of perfluorooctanoic acid (PFOA) in the environment and human surroundings, as well as its toxic action and its selected short-chain analogs—perfluorohexanoic acid (PFHxA) and perfluorobutanoic acid (PFBA)—has been described. These substances belong to a group of polyfluoroalkyl substances (PFASs) that are widely represented in various compartments of the environment, including the air, water and soil. They are also present in food, drinking water and dust, which are the main sources of exposure for humans to these substances. Due to their physico-chemical properties, PFASs are resistant to degradation in the biosphere and therefore effectively accumulate in biota, including humans. PFOA has been produced for decades, but due to its toxicity, it was subsequently replaced by PFASs with a shorter chain, including PFHxA and PFBA, whose presence in the environment, as well as risk of human exposure and toxicity, has been poorly investigated. It has been proven that PFOA has hepatotoxic and endocrine-disrupting activities, as well as prooxidative, immunotoxic, epigenetic and carcinogenic potential. Previously conducted studies have shown that PFHxA and PFBA are less toxic than PFOA; nevertheless, additional extensive studies should be conducted in order to determine the environmental and toxicological status of these compounds.

## 1. Introduction

Industrial activity negatively affects the environment. One example is the introduction of xenobiotics intentionally or as by-products formed during the production of other chemical substances. Hazardous substances are those that negatively influence human health and accumulate in the environment and biota, including humans. Examples of such chemicals are per- and polyfluoroalkyl compounds (PFASs). First introduced in 1949, PFASs became widely used chemicals in industry and were used in various food contact materials, including anti-stick kitchen appliances and food wrapping [1].

PFASs are a group of synthetic chemicals containing a carbon chain, in which all (per-) or parts (poly-) of the hydrogen atoms are replaced by fluorine atoms. Moreover, these substances have specific functional groups at the end. Taking into consideration the number of carbon atoms contained in the chain, PFASs can be divided into long-chain (containing at least seven carbon atoms) and short-chain compounds (containing four to six carbon atoms). With respect to their characteristic structure, PFASs have amphiphilic properties (simultaneously hydrophobic and hydrophilic). These substances are extremely persistent in the environment; are physically, chemically and biologically stable; are resistant to degradation; and accumulate in living organisms, including humans [2].

Fluorinated tails of PFASs have both hydrophobic and oleophobic properties, which include unique properties with numerous commercial applications, such as use as powerful surfactants that decrease surface tension much more effectively than hydrocarbon surfactants, which is why firefighting foams use these substances. Oleophobic (anti-fat) properties have led to the use of PFASs products in the food container industry, and their hydrophobic/oleophobic nature has made them popular in the stain-resistant and anti-stick cookware industries. Due to their unusual characteristics, PFASs are also used in food packaging, waterproof clothing, non-stick cookware, textiles, cosmetics, pesticides and pharmaceuticals [3,4].

Food and drinking water are the most important matrices from which PFASs enter the body; however, they also penetrate the human organism through dust inhalation or inhalation of small particles, from which they are absorbed. Skin is the least important route of PFASs exposure. The body cannot metabolize PFASs, and their removal from the human organism is slow [5,6,7,8].

Numerous research works have studied the toxic effects of PFASs on the human body. An analysis of the literature shows that PFASs contribute to an increased risk of cancer; disrupt the function of internal organs, e.g., the liver or kidneys; and cause endocrine and metabolic disorders, including insulin resistance, hypertension and obesity [5].

Taking into account the common occurrence of perfluorooctanoic acid (PFOA) in the environment and its harmful effect on living organisms, this compound was entered into the list of persistent organic pollutants under the Stockholm Convention, and additional restrictions on its use were established by 2020. In turn, the US Environmental Protection Agency (US EPA) decided to withdraw PFOA from US territory by 2015 [9].

As a result, more and more manufacturers started to replace PFOA with short-chain alternatives (containing up to six carbon atoms). Short-chain substitutes show similar properties to long-chain substances, as they are characterized by strong amphiphilic properties, stability and durability. Examples of alternatives to PFOA are perfluorohexanoic acid (PFHxA) and perfluorobutanoic acid (PFBA). Researchers have also developed substitutes without fluorine atoms, including nanomaterials, nanoparticles and dendrimers, but these substances do not have as many useful properties as short-chain PFASs [10]. Despite the successive elimination of PFOA, it is still found in considerable concentrations in human samples. Similarly to PFOA, its shorter-chain substitutes have been detected in serum, tissue samples and urine. It was also proven that PFOA is present in the highest amounts among PFASs in serum and urine, which demonstrates its strong ability to bioaccumulate. Moreover, high amounts of PFOA and its substitutes have been found in human breast milk and human tissues, such as the lungs, kidneys, liver and brain. The occurrence of these compounds in the human organism is responsible for the development of various disorders and adverse effects on intestinal flora.

With respect to the results of studies concerning the harmful effects of PFASs on living organisms, the European Food Safety Authority (EFSA) established a weekly tolerable intake sum for PFASs of only 4.4 ng/kg of body weight per person [2].

PFOA substitutes have been introduced into the market as potentially safer compounds [11]. However, there is still too little scientific research on their environmental occurrence, human bioaccumulation, and toxicity and the molecular mechanisms of their action.

This review aims to compare long-chain PFOA with its short-chain analogs (PFHxA and PFBA) in terms of environmental occurrence, bioaccumulation and toxicity, with particular emphasis on whether these substitutes can be considered safer alternatives.

## 2. Chemical and Physical Properties

Perfluorooctanoic acid (PFOA) and its short-chain substitutes (i.e., PFHxA, PFBA) are man-made chemical compounds with a characteristic structure, in which all hydrogen atoms in the carbon chain are replaced by fluorine atoms (Table 1).

PFOA, PFHxA and PFBA have been classified as a perfluoroalkyl acids (PFAAs), because they have a hydrophilic carboxyl group at the end of their molecule. Moreover, the bond that links carbon and fluorine is very strong and stable [3,12,13,14,15,16,17]. The physiochemical properties of the discussed compounds are presented in Table 2.

PFAAs are formed as a result of environmental degradation and metabolic processes from other related per- and polyfluoroalkyl substances (PFASs), as well as precursor compounds [11,12,14,16]. For instance, fluorotelomer carboxylic acids (FTCAs) present in landfill are often degraded to PFASs with a lower number of fluorine atoms, and mainly PFHxA and PFBA [19].

PFOA, PFHxA and PFBA are weak acids that can partially dissociate in aqueous matrices to perfluorooctanoate ions (C7F15COO-), perfluorhexanoate ions (C5F11COO-), perfluorobutanoate ions (C3F9COO-) and hydrogen ions (H+), respectively [20,21]. PFOA is comparatively stable in normal environmental conditions, and is persistent in the environment, mainly in respect to its resistance to deterioration processes. Nevertheless, degradation of PFOA appears under exposure to high temperatures or UV irradiation [22].

## 3. Usage

In respect to unique properties of PFOA and other PFASs, such as stability, durability and lowering of surface tension, these substances are used as ingredients in firefighting foams (aqueous film-forming foams, AFFFs), textiles (membranes for waterproof and breathable clothing), electronic devices, semiconductors, cosmetics (body creams), pesticides, automotive waxes, and sports articles (ski waxes). They are also used in paints, additives on the surface of non-stick cookware and containers [23], and textiles and upholstery (impregnation spray for textiles) [24]. Moreover, they are used in floor covering, including nylon carpets, floor polish and laminated plastic floor covering [25,26].

## 4. Occurrence in the Environment and Human Surroundings

PFASs are named “perennial chemicals” because they show high accumulation and are chemically stable. PFASs have been found in rivers and marine ecosystems, and are implicated in hydrological cycles. In respect to their accumulation, when they reach a food chain, their content in food products and water rises [27,28]. The prevalent exposure routes of PFASs to a human population are consumption of contaminated food and polluted water, while inhalation of contaminated air (PFASs are adsorbed on aerosols and dust particles in indoor and outdoor environments) is less important [29] (Table 3 and Table 4).

### 4.1. Food

Based on the literature data, food comprises the highest concentrations of PFASs. PFOA has been found in fish (in perch from 5.22 to 67.8 ng/g; in eels from 5.73 to 68.8 ng/g), and PFHxA from 1.92 to 41.1 ng/g, crustaceans (PFOA from 8.56 to 157 ng/g, PFHxA from not detected (ND) to 3.77 ng/g and PFBA from ND to 2 ng/g, eggs (PFOA from 0.05 to 4.83 ng/g, while mean PFBA concentration was 81.4 ng/g and PFHxA was 0.82 ng/g), meat (from 8.78 to 12.1 ng/g for PFOA, from 0.06 to 3 ng/g for PFBA and from ND to 2.9 ng/g for PFHxA), seafood (from 2.33 to 54.6 ng/g for PFOA and from ND to 3.77 ng/g for PFHxA), and offal (from 2.15 to 283 ng/g for PFOA, from ND to 20 ng/g for PFBA and from ND to 5.14 ng/g for PFHxA). Contamination of food with PFASs is generally associated with multiple environmental pathways, such as emissions from industrial and municipal sources, which leads to their persistence in water and soil and subsequent transfer and bioaccumulation in the food chain [37,39,40,41,47,49]. The European Food Safety Authority (EFSA) has established the tolerable daily intake (TDI) of PFOA at 150 ng/kg body weight [42]. This value was based on the observed adverse effect level that was estimated for 0.06 mg/kg b.w./day. For instance, in Taiwan, the exposure to PFOA associated with consumption of contaminated food is 85.1 ng/kg b.w./day [43]. It is worth noting that PFASs, including PFOA are widely used in materials that are in contact with food. For instance, PFASs are used in materials, such as kitchen accessories with non-stick surfaces, fast food packaging, and microwaveable popcorn packaging, which may lead to the migration of these substances into food [43]. It has been proven that this migration rises along with the increase in temperature, a longer contact time with food, and the presence of emulsifiers in the food. The degree of migration is compound-specific; nevertheless, long-chain compounds, such as PFOA show faster migration than short-chain substitutes [44]. Therefore, the European Union has established maximum PFOA levels in selected food products, in which its concentrations have been determined to be the highest (Table 5). The discussed findings have confirmed that diet constitutes a significant route of human exposure to PFASs. The presence of these compounds both in food products and in food-contact materials indicates the complex nature of consumer exposure. Due to the ability of PFASs to bioaccumulate and their persistence in the environment, continuous monitoring of their concentrations in food and development of strategies aimed at reducing population exposure to these substances are necessary.

### 4.2. Water

PFOA and its short-chain analogs enter the body through ingestion of contaminated drinking water and such contamination usually occurs near industrial plants. Based on the literature data, PFOA enters groundwater through the soil, as well as migrating from river or well water [49]. Surface and groundwater pollution by PFOA is common, while industrial discharges, firefighting facilities, military operations, factories through leaking pipelines, and transportation have been considered as the main sources. The maximum PFOA concentrations determined in surface water were very different, such as ND–10.7 ng/L in Thailand, 1.6–73 ng/L in Canada, ND–223.8 ng/L in China, and ND–522 ng/L in Sweden. In groundwater, PFOA levels are also variable. For instance low concentrations of PFOA were detected in groundwater in Thailand (ND–34.96 ng/L), and high concentrations were found in China (ND–2510 ng/L), Canada (ND–3260 ng/L), Sweden (ND–4470 ng/L) and the USA (ND–24 µg/L). Generally, PFOA concentrations in drinking water are considerably lower than those found in groundwater, ranging from ND levels to approximately 4300 ng/L. Reported concentrations reached ND–7.6 ng/L in Canada, ND–9.7 ng/L in China, ND–16.5 ng/L in Thailand, ND–100 ng/L in Sweden, and ND–4300 ng/L in the USA. Similar concentrations have been determined for PFBA, e.g., 1.3 ng/L in Spain, 17.87 ng/L in China, and up to 9 ng/L in the USA. The highest concentration of PFHxA in drinking water was detected in the USA—1400 ng/L [28,29,42,43,47]. The differences in PFOA concentrations are likely related to the intensity of industrial activities and the presence of local PFASs emission sources, such as fluorochemical plants, airports and firefighting facilities [30]. In surface water in Bangladesh (industrial centers of Dhaka), PFBA, PFHxA and PFOA were detected at low concentration ranges from 1.0 to 7.5 ng/L for PFOA, from 1.6 to 15 ng/L for PFBA and from 1.8 to 8.1 ng/L for PFHxA. The detected PFBA, PFHxA, and PFOA concentrations in Dhaka surface waters were primarily driven by direct releases discharged from urban and industrial wastewater and the use of PFAS-containing products. Additional inputs arose from precursor degradation, while PFOA was also released from imported products and insufficiently treated effluents [31]. The frequent detection and wide concentration ranges of PFBA, PFHxA, PFOA, and perfluorooctanesulfonic acid) PFOS in Canadian freshwater sites are primarily driven by their widespread sources of release, including extensive historical and current industrial production and use of PFAS-containing products, as well as intentional substitution of long-chain PFASs (e.g., PFOA and PFOS) with short-chain alternatives, such as PFBA and PFHxA. These inputs are further reinforced by continuous emissions from wastewater treatment systems and persistent releases from legacy contaminated sites [32]. These results have shown that intensive implementation of short-chain analogs replaces those of PFASs with longer chains.

Similar results were achieved in the study of distribution of 23 PFASs in groundwater and surface water samples taken in urban areas in Brazil (Porto Alegre) [50]. It was found that the PFOA range in surface water was from 4 to 8 ng/L, PFHxA from 1 to 3 ng/L and PFBA from 0.2 to 2 ng/L, while PFOA was the most commonly detected. Moreover, the authors observed that concentrations of PFASs were higher in tributary waters than in larger water bodies. The occurrence and concentrations of PFASs were primarily driven by direct inputs from their sources of release, including urban wastewater effluents, industrial discharge, and runoff from areas where PFAS-containing products were used. Additional contributions come from diffuse emissions across urban environments and from long-term legacy contamination resulting from historical production and widespread use of these compounds. In China, total concentrations of PFASs were up to 260 ng/L in the Huang He River mainstream and 1526 ng/L in its tributaries, which showed the importance of dilution process and subsequent population exposure. Higher PFASs concentrations in tributaries compared to main river sections reflects dilution effects in larger water bodies and increased local inputs from surrounding anthropogenic sources, leading to elevated exposure in smaller catchments [51]. The study conducted in 2019 on surface water and sediments in Melbourne (Australia) showed that among PFAAs, PFOA (ND–13 ng/L) was the most commonly determined in urban areas, whereas PFBA (ND–5.7 ng/L) was the most frequently detected in rural areas [52]. The authors excluded contamination from areas near the airport, where the total concentration of 33 PFASs in surface water was 4261 ng/L. They also showed that the median total concentration of 33 types of PFASs in surface water was 63.5 ng/L, while the highest average concentrations were noted for PFBA (11.3 ng/L), PFHxA (9.2 ng/L) and PFOA (8.3 ng/L) with the most frequently detected being PFOA.

In another study, drinking water contamination by a fluoropolymer production plant was observed in Huantai County, China. PFOA was the prevalent reagent in the plant, which discharged it into Xiaoqing River in an amount of 160 kg/day. As a result, high concentrations of PFOA were detected in drinking water (5.95–19.3 ng/L), as well as in groundwater (up to 104 ng/L). Moreover, PFOA was detected in vegetables from local suppliers, with high concentrations in the range of 65–825 ng/g in wedge onion pseudostems, 200–825 ng/g in wedge onions, 900–2678 ng/g in celery, and 1000–5303 ng/g in carrots [53]. PFOA contamination in water and soil near the Huantai fluoropolymer plant likely resulted from direct industrial discharges into the Xiaoqing River and subsequent environmental dispersion [54]. One of the countries using the monitoring of xenobiotics to assess their human exposure dynamics is Slovenia. The program has been coordinated by the Slovenian Chemicals Office since 2007. Runkel et al. [29] carried out systematic human biomonitoring (HBM) study of PFASs and found PFOA in all samples tested (0.87–1.16 ng/mL), whereas the highest concentration of PFOA (1.16 ng/mL) was determined to be in the population inhabiting the Ravensko region. The source of PFOA contamination were diffuse anthropogenic emissions related to the widespread occurrence of PFASs in the environment, including both long-term background contamination (legacy contamination) and migration of these compounds from PFAS-containing products into water resources.

As mentioned above, PFASs have often been found in drinking water. These compounds have been frequently determined in drinking water treatment plants that use traditional treatment methods that fail to remove these substances. Worldwide, it has been evaluated that PFOA levels in tap water are in the range from a few ng/L to as high as a few µg/L. Nevertheless, according to EPA, the concentration of PFOA in prevalent tap waters is very low—about 0.004 ng/L. Highly advanced technologies may provide better results from the removal of PFASs, but this effect also depends on PFASs’ properties and their concentration in raw water [30].

In 2018, the Agency for Toxic Substances and Disease Registry (ATSDR) established the admissible PFOA concentration in drinking water as being 11 ng/L. In 2022, the US Environmental Protection Agency (US EPA) proposed much stricter standards, in which the level of PFOA in drinking water should not exceed 0.004 ng/L [35]. However, in March 2023, the US EPA changed this limit to 4 ng/L [30].

### 4.3. Air and Surface Snow

PFASs levels are not systematically monitored in the air or snow. Björnsdotter et al. (2021) [38] investigated seasonal levels and distribution of short-chain PFAAs, such as perfluoropropanoic acid (PFPrA) and PFBA in snow cover on the Spitsbergen island (Norwegian Arctic). They detected PFPrA (0.79–16 ng/m^2^) and PFBA (0.19–170 ng/m^2^) in most of the studied samples. It must be noted that PFPrA and PFBA were detected in samples collected from the locations, in which their occurrence can only be connected with long-range processes, such as geographic migration from the cities, in which they are produced and used. Moreover, positive association was found between the flux of these substances and solar radiation. In another study conducted near Fayetteville (North Carolina, USA), the presence of PFASs was noted in the air near a fluoropolymer manufacturing facility [33]. It was shown that among the short-chain substances, PFBA (0.1–0.7 pg/m^3^) and PFHxA (0.3–1.2 pg/m^3^) were determined in higher levels than other analyzed PFASs.

### 4.4. Dust

Many studies have proven that PFASs have an affinity for atmospheric particles, and therefore they can migrate long distances: for instance, from cities or factories to distant areas without industrial development. Such contamination is associated with both point sources (particular area of pollution) and the influence of atmospheric factors [28].

Taking into account that 90% of human population’s time is spent indoors (homes, schools, workplaces), dust and other kinds of particles are essential sources of human exposure to PFASs [36]. This exposure is linked to processes that release these compounds from everyday products (e.g., clothing, cleaning products, furniture) and leads to their adsorbtion on dust particles’ surfaces. PFOA was determined in 93% of samples of household dust, which were collected in Sweden (median concentration was 13 ng/g) [45]. Alternatively, a research study conducted in China (Tianjin) showed that PFOA and PFBA were dominant substances in dust samples collected from homes among 23 tested PFASs (PFOA and PFBA were determined in all analyzed samples). It was also noted that the median PFBA concentrations were 165 pg/m^3^ and 271 pg/m^3^ in hotels and homes, respectively [46]. The study conducted in the Thessaloniki (Greece) showed a range of PFHxA in dust trapped by a central air conditioner (i.e., restaurants and electronics shops) in concentrations ranging from 3.6 to 72.5 ng/g (presence in 85% of all analyzed samples), whereas 90–95% of samples consisted of PFOA in the significantly higher concentration range, from 10 to 653 ng/g [55]. The main sources of PFASs contamination in indoor environments is diffuse emissions from PFAS-containing consumer products and building materials, including textiles, furniture, electronics, and surface coatings. These emissions led to the release of PFASs into indoor air and their subsequent accumulation in settled dust and filtration systems, with additional contribution from background environmental contamination via atmospheric transport and deposition from outdoor sources.

## 5. Human Biomonitoring

PFOA, PFHxA and PFBA have been determined in the serum of humans that have been environmentally or occupationally exposed. PFOA has been repeatedly determined in humans that have been environmentally exposed, including serum (0.006–38.5 ng/mL) [56], urine (mean 7.3 ng/mL) [57], and in a high concentration in women’s milk (mean 336 ng/mL) [58]. Due to the hydrophobic properties of PFOA, it is also accumulated in kidney (mean 1.5 ng/g), liver (mean 4 ng/g) and lungs (mean 12.1 ng/g) [59]. In humans that have been occupationally exposed, high concentrations of PFOA were detected in full blood (up to 535 ng/mL) [60].

Generally, PFHxA has been found in comparable or higher concentrations than PFOA in human tissues. PFHxA was detected in the serum of humans that have been environmentally and occupationally exposed in concentrations ranging from 0.07 to 100 ng/mL [61,62]. Moreover, significant concentrations of PFHxA were determined in the liver (mean 68.3 ng/g), brain (mean 141 ng/g) and lungs (mean 207 ng/g) [59].

Interestingly, PFBA has been found in the highest concentrations among the discussed PFASs in kidneys (mean 263 ng/g), and particularly in the lungs (807 ng/g) of humans that have been environmentally exposed [59]. Moreover, PFBA was determined in the brain (mean 1.4 ng/g) and the liver (mean 3 ng/g) [59]. In the serum of environmentally and occupationally exposed humans, PFBA has been determined in the range from 0.008 to 2.5 ng/mL and from 3.7 to 5.4 ng/mL, respectively [63,64].

Detailed results of human biomonitoring studies of PFOA, PFHxA and PFBA are presented in Table 6 and Table 7.

## 6. Toxicity

The accumulation of PFOA and other PFASs is associated with their ability to bind to circulating serum albumin, through which they are transported to the liver and other organs, where they accumulate and cause damage [68]. Additionally, these substances can disrupt endocrine function by changing estrogen and androgen receptor activity and influencing thyroid hormone homeostasis [69]. Rodent studies have shown that PFASs also change glucose levels [70], increase leptin and insulin concentrations, and are potentially involved in weight gain [68]. Other adverse effects include liver, pancreatic, and breast cancers; abnormal protein metabolism; and hepatomegaly [47,71,72,73].

It is worth pointing out the very important role of signaling pathways in the development of various complex disease processes. It has been proven that dysregulation of cell signaling mechanisms affects gene expression and epigenetic toxicity, resulting in abnormal cell growth in tissue, resulting in the growth and development of pathologies, such as cancer, as well as inflammation and immunotoxicity.

Not all compounds classified as PFASs exhibit the same toxicological potential. Fluoropolymers, such as polytetrafluoroethylene (PTFE), have a high molecular weight, are characterized by exceptional chemical stability, and show very low water solubility. In respect to these properties, they are not bioavailable, which means that they do not cross biological membranes, are not absorbed by the organism, and have no potential for bioaccumulation. Therefore, fluoropolymers have been classified as so-called Polymers of Low Concern (PLC). Alternatively, low-molecular-weight PFASs, such as PFOA, show a different category. These substances are significantly more mobile in the environment and can be accumulated in the human organism through dietary intake, inhalation, or dermal contact. Moreover, they have the capability of accumulating in tissues and have long biological half-lives that increase the risk of adverse health effects [74]. Schlummer et al. [75] showed that heating PTFE surfaces may result in the emission of trace amounts of perfluorocarboxylic acids (PFCAs), including PFOA. The emission was strongly temperature-dependent and increased considerably when the surface was overheated above 260 °C. Importantly, the authors concluded that under temperatures corresponding to normal cookware use, the amounts of released compounds were very low and did not represent a significant source of consumer exposure.

### 6.1. Hepatotoxicity

PFASs accumulate mainly in the liver, therefore inducing hepatoxicity. PFASs such as PFOA have been shown to induce liver damage in animal models, such as mice, rats and monkeys, showing hepatotoxicity as an important toxicological activity [76,77,78].

In an in vitro study, Amstutz and co-workers (2022) [79] assessed the effect of PFASs with different carbon chain lengths and functional head groups (0–800 µM) on the viability of HepG2 cells and reactive oxygen species (ROS) formation. They noticed that the cytotoxic potential of PFASs, as well as ROS formation, was dependent on chain length, as stronger effects were observed for PFOA and weaker for PFHxA and PFBA, which showed similar toxic potential in tested cells.

The results achieved in animal studies have shown that PFASs induce different alterations in the liver, including changes in metabolism, as well as in lipids and protein levels, and cause liver damage. For instance, Das et al. (2017) [80] observed a dose-dependent increase in hepatocellular hypertrophy, and liver weight in response to PFASs, including PFHxA. The liver has been considered one of the main targets of PFHxA toxicity. A sub-chronic toxicity study showed that PFHxA could induce centrilobular hepatocellular hypertrophy in rats [81], while exposure to a sub-lethal dosage of PFHxA altered liver cell metabolism in zebrafish [82]. Similarly, in an experiment conducted on Sprague–Dawley rats, PFOA significantly changed the alanine transferase (ALT) level and induced liver edema and liver toxicity by mechanisms connected with specific protein denaturation [83].

Peroxisome proliferator receptor alpha (PPARα) is a member of the nuclear hormone receptor superfamily. The central physiological role of PPARα is as a ligand-activated transcription factor whose target genes encode enzymes and proteins implicated in fatty acid transport and catabolism. The activation of PPARα by PFASs, including PFOA, PFHxA and PFBA has been recognized as the primary mechanism of action in rodent hepatocyte-induced proliferation. For instance, Foreman et al. (2009) [84] evaluated the role of PPARα in mediating hepatotoxic effects of PFBA (350 mg/kg/day) in PPARα null mice and a mouse line expressing human PPARα in the absence of mouse PPARα. The authors showed that PFBA modulated lipid metabolism, increased liver weight and caused hepatocyte hypertrophy in wild-type and PPARα humanized mice. They also noticed that PFBA induced hepatocyte focal necrosis with inflammatory cell infiltrate only in wild-type mice. As a result, they concluded that PFBA activated both the mouse and human PPARα, but a species difference existed in the hepatotoxic response to this chemical. In another study, PFBA induced peroxisome proliferation and increased peroxisomal fatty acid oxidation in rat liver, which are biomarkers of PPARα activity [85]. In addition, when pregnant Kunming mice were exposed to PFOA, it reduced the growth and development of the pups and induced liver damage, which altered the secretion of enzymes implicated in PPARα-induced fatty acid oxidation, which led to hepatic bleeding, local necrosis, enlargement of hepatocytes, and depletion in histone acetylation [86].

PFASs are activators of PPARα, but biological effects of these chemicals are also probably mediated by other factors. In order to evaluate this hypothesis, male wild-type and PPARα null mice were administrated by oral gavage with PFHxA (3 or 10 mg/kg/day) for 7 days, and expression of liver genes was assessed by full-genome microarrays. Using data available through a microarray database, PFHxA expression of genes profiles were not found to reveal considerable similarity to profiles from mouse tissues exposed to agonists of the constitutive activated receptor (CAR), estrogen receptor α (ERα), and PPARγ. In conclusion, a negative relationship was found in all treated wild-type mice, along with similar but muted effects in PARPα-null mice exposed to PFHxA. In a recent study, Robarts et al. (2024) [87] studied human-relevant mechanisms of PFASs action, including PFOA-induced hepatic effects using FRG liver-chimeric humanized mice with livers repopulated with functional human hepatocytes. Male FRG humanized mice were treated with low doses of 0.145 mg/L of PFOA in drinking water for 28 days. They noticed that PFOA induced a decrease in the total serum cholesterol and LDL/VLDL fractions, as well as causing significant hepatocyte proliferation. RNA-sequencing with alignment to the human genome showed a total of 162 differentially expressed genes as a result of PFOA exposure. Moreover, upstream regulator analysis showed that PFOA caused activation of p53 and inhibited of androgen receptor and nuclear receptor subfamily 1 group D member 1 (NR1D1), which is a transcriptional repressor that is important in the circadian rhythm. Further biochemical studies confirmed NR1D1 inhibition. The authors concluded that new human-relevant molecular mechanism of PFASs exists, including their previously unknown effect on circadian rhythm.

Several studies have shown the effect of PFASs on liver function, metabolism and damage. For instance, PFOA (5 mg and 10 mg/kg) induced steatosis, increased fatty acid translocase and lipoprotein lipase expression, and depleted oxidation of fatty acids, which led to a reduction in energy production in mature 8-week old male mice [88]. In another study, Atemma and co-workers (2022) [89] administrated PFOA at a dose of 0.3 mg/kg to male wild types and nuclear PPARα played an essential role in the transcriptional regulation of lipid homeostasis [90]. They noted that PFOA disrupted hepatic metabolism in the studied animals. PFOA also improved glucose and insulin tolerance but reduced body weight and raised liver weight in wildtype and PPARα mice. Moreover, PFOA, in a PPARα-dependent manner, depleted plasma cholesterol and triglycerides. Specifically, 88% of the regulation of gene expression by a high dose of PFOA in mouse liver was dependent on PPARα. In conclusion, the authors suggested that the effects caused by PFOA were mediated by multiplying nuclear receptors. In another study, the exposure to PFOA increased blood cholesterol and triglycerides levels, as well as causing non-alcoholic fatty liver disease in humanized PPARα mice fed with an American diet [91]. An extended study was conducted by Stoffels et al. (2023) [92], who studied a global lipidomic analysis on the liver of PFOA-exposed mice. Among all hepatic lipids detected, the levels of more than 350 were statistically raised or depleted. It was also noticed that the levels from many lipid species of various lipid classes, mostly phosphatidylethanolamine, phosphatidylcholine and triacyglycerols, were significantly changed. Furthermore, subsequent lipidomic analysis highlighted the pathways that were substantially affected by PFOA, with the glycerophospholipid metabolism being the most changed, and the alterations in the lipidome network, which connects all the lipid species together. In conclusion, this analysis showed heterogeneous distribution of the altered lipids by PFOA, showing different areas of lipid expression connected to PFOA localization.

Schlezinger et al. (2020) [93] tested the hypothesis that PFOA exposure at a human-relevant level dysregulates gene expressions that control cholesterol homeostasis in livers of mice that express human PPARα (hPPARα). Female and male hPPARα and PPARα null mice were fed with an American diet and treated in drinking water (8 μM) for 6 weeks. The authors noticed that PFOA raised liver mass, which was connected to histologically evident lipid accumulation. Moreover, PFOA increased the serum cholesterol level and caused PPARα and constitutive expression of the androstane receptor target gene in the liver. Moreover, gene expression in four pathways which regulate cholesterol homeostasis was observed. The authors also noticed that PFOA depleted expression of 3-hydroxy-3-methylglutaryl-CoA reductase (Hmgcr) in a PPARα-dependent manner, as well as reducing the expression of low-density lipoprotein receptor (Ldlr) and cholesterol 7 alpha-hydroxylase (Cyp7a1) in a PPARα-independent manner. They concluded that the obtained results provided new insight into the effect of PFOA on cholesterol regulation in the liver and the role of hPPARα.

An interesting study was conducted by Jiang et al. (2021) [94], who studied the transcriptomic, proteomic, and metabolomic parameters in mice exposed to PFHxA. Among 24,893 RNAs and 2246 proteins, 566 transcripts and 238 proteins were substantially changed in mice exposed to PFHxA. The authors identified processes which were implicated in liver damage, such as alterations in biosynthesis of fatty acids, as well as pathways of degradation, such as apurine metabolism (depletion levels of xanthine and uric acid) and glutathione (GSH) metabolism, that might be triggered by the PPAR signaling pathway. They also observed that PFHxA caused oxidative stress, because an increase in SOD activity and a decrease in GSH level were noticed.

It was shown that high doses of PFOA can induce hepatocellular adenomas in rats [95]. It is known that the inhibition of gap-junctional intercellular communication (GJIC) is needed, however insufficient the step of tumorigenesis is, and it is therefore a typical response of cells to tumor promoters, oncogenes, growth factors, and nongenotoxic carcinogens, including peroxisome proliferators [96]. Perfluoroalkanoates, such as PFOA have been shown as peroxisome proliferators that trigger hepatomegaly and hepatocarcinogenesis in rodents, and have been recognized as classic non-genotoxic carcinogens inhibiting GJIC in carcinogenesis.

Upham et al. (2009) [97] noted that PFOA inhibited GJIC and triggered hepatomegaly in rat livers. Although the serum biochemistry of liver enzymes did not reveal any cytotoxic response to this compound, in vitro analysis of mitogen-activated protein kinase (MAPK) showed that PFOA activated the extracellular receptor kinase (ERK). The authors also noticed that the inhibition of GJIC, in vitro by PFOA, depended on ERK and phosphatidylcholine-specific phospholipase C (PC-PLC) activation in the dysregulation of GJIC in an oxidative-dependent mechanism. In conclusion, they stated that in vitro analysis of GJIC as an epigenetic marker of tumor promoters can predict the in vivo activity of PFOA, which can dysregulate GJIC via ERK and PC-PLC [97].

The gut microbiome functions as a ‘metabolic organ’ that interacts with the host for a mutually beneficial coexistence. Perturbation in gut microbiome can disrupt gut barrier permeability, leading to production of translocated bacteria and leakage of gut-derived products that reach the liver through the portal venous system. This results in inflammation, oxidative stress and liver diseases [98], suggesting a ‘liver–gut-axis’ existence. In the present study, the impact of subacute (30 mg/kg) and subchronic doses (3 mg/kg) of PFOA administrated orally for 14 days on liver and gut microbiota in C57BL/6J mice was evaluated. It was shown that subchronic and subacute exposure to PFOA induced inflammation of the liver, changed antioxidative homeostasis and caused liver histological abnormalities, such as hepatomegaly that caused liver damage. Moreover 16S rRNA sequencing analysis revealed that mice exposed to subacute doses of PFOA had changed the amount of intestinal flora, such as Dehalobacterium and Bacteroides genera, which contributed to the inflammation of the liver and oxidative stress. Moreover, the exposure to subchronic doses of PFOA mostly caused a reduction in commensal probiotics, such as Lactobacillus and Bifidobacterium genera that are potentially beneficial when liver damage occurs. In conclusion, the authors suggested that liver damage caused by PFOA was partly related to the gut microbiota dysbiosis.

In occupational surveys, the association between human exposure to PFASs and higher risk of liver damage and disfunction have been observed. Choi et al. (2022) [99], in a review and meta-analysis, evaluated the relationship between exposure to PFOA and hepatic diseases determining alanine aminotransferase (ALT), which is the standard screening tool for detecting acute hepatic injury. The results showed that people exposed to PFOA had elevated ALT activity in comparison to individuals not exposed to this substance. Nevertheless, the authors of the study concluded that due to the limited number of individuals tested in the analysis, it is premature to suggest that PFOA exposure is connected to development of hepatic diseases in humans. Nevertheless, Sen et al. (2022) [100] found a positive association between PFASs, including PFHxA levels in serum and nonalcoholic fatty liver disease (NALFD)-associated lipid changes in human livers. In another study, the association between the PFOA level in serum and liver enzyme activities was analyzed using 9523 Americans aged 20 years or older (National Health and Nutrition Examination Survey). The authors suggested that alterations in liver enzymes, including ALT, aspartate aminotransferase (AST) and gamma-glutamyl transferase (GGT) were associated with PFOA exposure [101]. Also, Sen et al. (2022) [100] investigated the effects of PFASs, including PFOA and PFHxA exposure, on liver metabolism in the human NAFLD cohort of 105 individuals (70 female and 35 male) in order to evaluate the metabolism of the liver, and in particular, the metabolism of bile acids. As a result, they noted upregulation of bile acids, triacylglycerols and ceramides, as well as an altered glucose level (insulin resistance) and amino acids’ (alanine, aspartate and glutamate) metabolism. In conclusion, they stated that human exposure to tested PFASs caused metabolic processes connected with NAFLD, and that the observed effects are different and generally stronger in females in comparison to males. To note, the study of Jin et al. (2020) [102] also showed that PFOA caused fatty liver in adults and non-alcoholic fatty liver in children.

Children are potentially more susceptible to toxicants, including PFASs. Seventy-four children with physician-diagnosed nonalcoholic fatty liver disease were recruited from Children’s Healthcare of Atlanta between 2007 and 2015 to evaluate metabolic dysregulation associated with PFASs, including PFOA and PFHxA. An integrative analysis showed a cluster of children with nonalcoholic steatohepatitis (NASH), which was characterized by raised PFASs levels and changed metabolite patterns, such as higher plasma levels of phosphoethanolamine, tyrosine, phenylalanine, aspartate and creatine, and depleted plasma levels of betaine, which proved the dysregulation of lipid and amino acid pathways associated with NAFLD pathogenesis. In conclusion, higher PFASs exposure was linked to more severe disease in children with NAFLD, and the authors of this study concluded that tested PFASs may be important toxicants contributing to NAFLD progression [102]. An interesting study of Stratakis et al. (2020) [103] showed that prenatal exposure to PFASs, including PFOA was linked to raised susceptibility to liver functions in children. They noted that increased PFOA levels in maternal blood were connected with higher concentrations of ALAT, AST and GGT in child serum. Moreover, substantial perturbations in amino acids leves and glycerophospholipid metabolism were observed.

Stronger associations between exposure to toxicants and adverse effects are usually observed in occupational surveys. In a study, 40 occupational workers from a factory in China and 52 control subjects from the general population were studied in an investigation on the potential health problems of occupational exposure to PFASs using mass spectrometry-based metabolomics analysis. It was noted that PFASs levels, including PFOA (0.57 µg/mL), PFHxA (2.15 µg/mL) and PFBS (0.22 µg/mL), were much higher in workers than in control individuals. In the study, 14 potential biomarkers were determined, and they were found to be linked to oxidative stress, fatty acid β-oxidation, and kidney injury. The authors concluded that the health effects of workers were associated with exposure to tested PFASs [104].

### 6.2. Endocrine-Disrupting Activity

The World Health Organization [105] defines endocrine disruptors (EDs) as “Exogenous substances that alter function(s) of the endocrine system and consequently cause adverse health effects in an intact organism or its progeny, or (sub)populations”. The studies have shown that perfluoroalkyl acids behave as endocrine disruptors. In their review, Borghoff and co-workers (2018) [106] analyzed data on the endocrine potential of PFHxA, evaluating estrogen, androgen, thyroid and steroidogenesis pathways as defined by WHO on different models, such as Japanese medaka, juvenile rainbow trout, chickens or reproductive parameters in northern bobwhite. They concluded that PFHxA did not show any endocrine potential in Japanese medaka, juvenile rainbow trout, chickens nor reproductive alterations in northern bobwhite, with no significant activity in rodent repeated-dose toxicity, life time cancer, or reproductive and developmental studies. The authors also pointed out that in studies of repeated doses in mammals, PFHxA did not significantly alter organs/tissues (weights or histopathology), such as testes, ovaries, thyroid, prostate, pituitary, mammary gland, or uterine. PFHxA also exhibited negative activity in vitro and in vivo for disrupting steroidogenesis. Only in vitro, weak or negative activity for thyroid (T) transport protein or activation of estrogen (E), androgen (A) or T receptors (T) was noted. Based on this analysis, the authors concluded that PFHxA did not induce alterations in endocrine activity in these models, and therefore it would not be characterized as an endocrine disruptor according to the WHO definition.

Thyroid hormones (THs) play crucial roles in the human endocrine system by regulating protein synthesis, energy metabolism, growth, and development. They also regulate the heart rate and blood pressure and activate hepatic lipolytic enzymes to regulate blood lipids [107]. Chengelis et al. (2009) [81] conducted a 90-day toxicity study, in which male and female Sprague–Dawley rats were treated with PFHxA by an oral gavage at a dose of up to 200 mg/kg/day. As a result of this investigation, no changes in endpoints showing endocrine disrupting potential, such as estrogen, androgen and thyroid receptors, or steroidogenesis pathways were observed. Similarly, a study conducted in Japanese medaka at aqueous concentrations of sodium or ammonium salt of PFHxA of 10 mg/L and 100 mg/L [108] did not show any significant differences in fecundity, fertility, survival or estrogen biomarker vitellogenin (Vtg) in comparison to control fish. Nevertheless, Wasel et al. (2021) [109] reported the developmental toxicity of PFASs in zebrafish and showed decreasing LC_50_ values with an increasing chain length for PFBA, PFHxA and PFOA.

In contrast, the studies have proven that PFOA can behave as an endocrine disrupter by altering the functions of growth and sex hormones, including activating the estrogen receptor (ER) and inducing ER-mediated transcriptions in cells [110]. For instance, an in vitro study showed that PFOA induced expression of estrogen-responsive genes based on different cell lines, such as the human CHO-K1 cell line and HepG2 cell line [111]. The ER transcriptional activation of PFOA was also noticed in different species in vivo, including rainbow trout (*Oncorhynchus mykiss*), in which induction of Vtg was noticed. In the same study, the authors observed a weak interaction of PFHxA with the estrogen pathway, showing a competitive binding affinity in rainbow trout to be <0.01% of E2; however, they concluded that the observed weak affinity did not translate to an in vivo response. In another study, Zhang et al. (2014) [112] treated male mice with PFOA (0–20 mg/kg/day) by oral gavage for 28 days. They observed that PFOA caused considerable damage to the seminiferous tubules and decreased progesterone and testosterone levels in the testis in a dose-dependent manner. Moreover, exposure to PFOA caused an increase in sperm quality. Using a quantitative proteomic approach, the authors determined 93 differentially expressed proteins in tested animals treated with PFOA at 5 mg/kg/day. Among determined proteins, insulin like-factor 3 (INSL3) and cytochrome P450 cholesterol side-chain cleavage enzyme (CYP11A1), as Leydig-cell-specific markers, were considerably reduced. The authors also assessed the expression patterns of CYP11A1 and related genes implicated in steroidogenesis in the mouse testis in detail. They found that PFOA, in a dose-dependent manner, depleted mRNA and protein levels of CYP11A1, and mRNA levels of 17β-hydroxysteroid dehydrogenase (17β-HSD). Additionally, an in vitro study showed a decrease in the progesterone level, which was accompanied by a reduction in the expression of CYP11A1 in cAMP-stimulated mLTC-1 cells. The authors concluded that exposure to PFOA can impair male reproductive function, possibly by altering testosterone levels, and CPY11A1 may be a key steroidogenic enzyme targeted by PFOA.

The mammary gland is particularly vulnerable tissue because of developmental endpoints, such as functional, milk protein gene expression, and developing neonatal and peripubertal structures. In the study of White et al. (2011) [113], one group of dams of mice was treated with 0, 1, or 5 mg of PFOA/kg/day during 17 days of gestation. Additionally, a second group of female mice was treated with 1 mg/kg/day during gestation and their F1 and F2 offsprings were continuously treated with a low concentration of PFOA (5 ng/mL) in drinking water. The authors found that gestational treatment with PFOA caused a delay in mammary gland development or/and lactational differentiation within three tested mice generations. Interestingly, chronic treatment of mice with low concentrations of PFOA in drinking water (relevant to its level in human water supplies) also changed mammary morphological development in tested animals. Zhao et al. (2012) [114] aimed to study the underlying mechanism of mammary gland development in various developmental stages in mice exposed to PFOA. Female 3-week old female wild mice were treated with PFOA at 2.5 mg and 7.5 mg/kg body weight for 2 weeks. The authors observed that PFOA considerably inhibited growth of mammary gland both in Balb/c and C57Bl/6 wild type mice, but not in C57Bl/6 PPARα knockout mice. The authors of the study also observed that PFOA caused a delay or absence of vaginal opening and induced lack of estrous cycling during the experimental period. Moreover, PFOA depleted the level of ovarian steroid hormonal synthetic enzyme and decreased expression of estrogen- or progesterone-induced mammary growth factors. The authors concluded that PFOA influences the ovaries, leading to mammary gland development, and that PPARα expression was a contributing factor in tested animals.

The endocrine-disrupting potential of PFOA has also been observed in occupational surveys. Jain et al. (2013) [115] determined the effect of PFASs, including PFOA on thyroid stimulating hormone (TSH), free and total thyroxine (FT4, TT4), free and total triiodothyronine (FT3, TT3), and thyroglobulin (TGN) in the general population of the USA, based on data from the National Health and Nutrition Examination Survey for the years 2007–2008. It was found that PFOA caused an increase in TSH and TT3 levels. Another epidemiological study assessed the association between PFOA concentrations in the general U.S. adult population and their current thyroid disease (data from the U.S. NHANES from 1999 to 2006) [116]. This study found that high concentrations of PFOA in serum were potentially associated with current thyroid disease. In other study, Tan et al. (2024) [117] evaluated association between the exposure of the elderly (n = 746, aged >60 years) from Taiwan to several PFASs, including PFOA, PFHxA and PFBA and thyroid hormone levels, such as TSH, thyroxine (T4), triiodothyronine (T3), FT4, and FT3 in plasma samples of tested subjects. Interestingly, they observed that the presence of PFBA was negatively correlated with the FT4 level, while for PFOA and PFHxA, the correlation has not been found. Generally, the authors observed that PFASs were associated with decreased TSH and FT4 levels and increased T4 and T3 levels, which suggested that those substances could cause thyroid-disrupting effects in the elderly population. Infants and children are particularly vulnerable to xenobiotics. Kim et al. (2016) [118] assessed the exposure levels of 16 PFASs in South Korean infant serum and correlated these levels with THs. A case group of infants suffered from congenital hypothyroidism. They observed a weak correlation between the concentration of PFOA and the level of thyroid-stimulating immunoglobulin (TSI) antibodies in the serum of tested individuals from the case group. In the study of Liu et al. (2020) [119], the association between PFASs, including PFOA and glucocorticoids (11-deoxycortisol, cortisol and cortisone) and two progestogens [progesterone, 17-hydroxyprogesterone (17OHP)] in the cord sera of 374 neonates (Wuhan, China) was analyzed. They found that PFOA was capable of significantly changing 11-deoxycortisol concentrations in cord sera. In another study, Liu et al. (2021) [120] assessed the associations between PFASs, including PFOA with estrogens, including estrone, estriol and estradiol levels in cord sera in newborns (n = 942, 2013–2014) in Wuhan, China. They found that there was a correlation between PFOA level and estradiol level in neonates. In the review study by 121. Ballesteros et al. (2017) [121], the associations between PFASs, including PFOA and TSH, T3, T4 levels or thyroid dysfunctions in pregnant women and children were assessed. The authors found positive associations between the PFOS level and TSH level measured in maternal blood.

### 6.3. Immunotoxicity

Studies have found several associations between immunotoxicity, including immunosuppression, hypersensitivity, and autoimmunity and PFASs exposure. The studies have also found significant inverse associations between serum PFOA levels and antibody responses to vaccines.

A recent in vitro study assessed the effect of PFBA, PFHxA and PFOA on cytotoxic and oxidative parameters in human peripheral blood mononuclear cells (PBMCs). It was found that PFBA, and more strongly PFOA, at 100 µg/mL and 200 µg/mL after 24 h of incubation decreased PBMCs viability, while PFHxA did not change this parameter even at 200 µg/mL. Moreover, PFOA and PFBA after 1 h, and particularly after 24 h of incubation significantly depleted the ATP level. The authors also noted that all studied PFASs at different concentrations caused oxidative stress and damage in PBMCs (0.1–50 µg/mL, 1 h incubation) because they increased ROS and reactive nitrogen species (only PFOA) levels, as well as causing damage to lipids and proteins in studied cells. Generally, most tested parameters were most strongly altered by PFOA, while PFBA caused stronger alterations than PFHxA [122].

In vivo studies of high, acutely toxic dietary doses of PFOA, up to 75 mg/kg/day resulted in suppression of antigen-specific immunoglobulin M (IgM) antibody production, splenic and thymic atrophy, and altered T-cell phenotypic distribution in male C57BL/6 mice [123]. In a TDAR (the T-Dependent Antibody Response assay) study with mice exposed to PFOA, an overall reduction in Th2 cytokines (significant: IL-5 and IL-13; non-significant: IL-4) and a mixed response for Th1 cytokines (significant reduction in IL-6, IL-12 and TNF-α) were observed by De Guise and Levin (2021) [124]. This showed a favorable Th1 balance and a general decrease in pro-inflammatory cytokines. The authors suggested a potential role for T helper (Th) cells in the immunotoxicity of PFOA. Significant dose-dependent changes in IgM level (which is mainly involved in early, primary immunity) in response to T-dependent antigens, such as sheep red blood cells (sRBCs) or horse red blood cells, were observed in acute and intermediate oral administration in C57BL/6J and C57BL/6N female mice, while the lowest adverse effect were observed for a dose of 3.75 mg/kg/day in mice exposed to PFOA in drinking water for 15 days [123,125]. By contrast, no effect on antibody responses was observed in Sv/129 rats dosed with PFOA in drinking water (30 mg/kg/day) for 15 days [123], suggesting strain differences in susceptibility to PFOA. Rats were less sensitive than mice, as no changes in IgM levels were noticed in these animals administered with PFOA (even at acutely toxic oral doses of up to 30 mg/kg/day) via gavage for 28 days [126]. Similarly, the exposure of male rats to PFOA (50 mg/kg/day) by gavage for 14 days did not significantly change the number of T cells, NK cells, or Th cells [127]. In a developmental mouse study, splenic T regulatory (Treg) number cells were reduced at the highest dose of PFOA at 2 mg/kg b.w./day, whereas isolated CD4+ cells from adult offspring, exposed via the dams to PFOA during gestation and through weaning, secreted lower amounts of the immunosuppressive cytokine IL-10 than cells from controls in males only. More recently, Ehrlich et al. (2023) [2] reported that in mice, a reduced number of thymocytes, splenic lymphocytes and marrow B-lymphoid cells decreased following exposure to PFOA (0.002% *w*/*w* in diet). Similarly, for the studies described above, Ehrlich et al. (2023) [2] concluded that PFOA may be recognized as an immunosuppressant in rodents, and pointed out that rats are more susceptible than mice to antibody immunosuppression induced by PFOA.

Several epidemiological studies have found associations between serum PFOA levels and diagnosis of asthma in children and adults, while a case–control study found significantly higher serum PFOA levels in asthmatic adolescents compared to adolescents without asthma [128]. Similarly, positive associations with two immune conditions were detected in two independent studies. The prevalence of self-reported asthma was significantly positively associated with residence in a PFOA-contaminated water district [129] and Taiwanese children [130]. A significant inverse association between the eosinophil count among non-asthmatic subjects, C-reactive protein (CRP), wheezing, eczema and PFOA level was found by food allergy and otitis media [131]. In the study of Lopez-Espinosa and co-workers (2021) [132], associations were found between peripheral white blood cell counts in a human population in the Mid-Ohio Valley, U.S. and PFOA level in drinking water. In this study, the PFOA level was positively associated with absolute lymphocyte count and the counts of T-cells, B-cells, and NK cells, whereas no significant associations were reported for changes in the percentages of B, Th and Tcytotoxic (Tc) lymphocyte subsets. The cross-sectional study of osteoarthritis (autoimmune condition) was based on 3731 adults with osteoarthritis and 45,701 without osteoarthritis who lived, worked, or attended school in one of six PFOA-contaminated water districts in the Mid-Ohio Valley and were enrolled in the C8 Health Project. The results of this project showed significant positive associations between increasing serum PFOA levels and osteoarthritis prevalence, while the observed association was limited to adults under the age of 55 years and those who were not obese [133]. Validated self-reported autoimmune diseases were also analyzed separately among the 3713 polymer plant workers. In this cohort study, ulcerative colitis and rheumatoid arthritis were reported more frequently [134]. Studies evaluating the immunosuppressive effects of PFOA have examined disease resistance and antibody responses. Granum et al. (2013) [135] conducted a prospective birth cohort study of mother–child pairs in Norway and found associations between a 1-ng/mL increase in the maternal perinatal plasma PFOA level and the number of episodes of the common cold and other respiratory tract infections, as well as the number of episodes of gastroenteritis with vomiting or diarrhea in the first three years of life and during the third year of children. Similarly, Granum et al. (2013) [135] found significant positive associations between a 1-ng/mL increase in PFOA level in maternal perinatal plasma and the number of episodes of the common cold. In a study of Wang et al. (2019) [47], 21 PFASs were identified in 198 Chinese women of childbearing age. In single PFASs, including PFOA, they were positively associated with Th1 and regulatory T-cell (Treg) cytokines, and negatively associated with Th2 and Th17 cytokines. In this study, the Bayesian Kernel Machine Regression (BKMR) model showed a significantly positive association between PFASs mixtures and TGF-β, as well as a negative association with IL-10. A deviation of the immune system from Th2 to Th1 cytokines has been implicated in pregnancy complications, such as recurrent miscarriage, preeclampsia and fetal growth restriction [47]. In their study, Knudsen and co-workers (2018) [136] studied the association between the sum of 15 PFASs, including PFOA and hematological markers in 189 Greenlandic pregnant women. The studied markers included white blood cells, lymphocytes, neutrophils and monocytes, which were significantly inversely associated with the sum of PFASs, suggesting an immunosuppressive potential of PFASs in pregnancy. Prenatal exposure to PFASs has been inconsistently linked to asthma and allergy, as well as the increased number of infections in early childhood. Impinen et al. (2019) [137] evaluated the association between exposure to PFASs, including PFOA of Norwegian Mothers (1270 women) and asthma, allergies and common infectious diseases in their children up to 7 years old. As a result, no association between PFOA levels and allergy and asthma were found. The authors also suggested that asthma and allergies in children may be linked to exposure to longer PFASs, as they and other scientists [138] observed such association for perfluoroundecanoic acid (PFUnDA) and perfluorotriodecanoic acid (PFTrDA).

Abraham et al. (2021) [139] showed considerable associations between the PFOA serum level and levels of vaccine antibodies against *Haemophilus infuenza* type b, tetanus and diphtheria in 101 healthy 1-year-old children. PFOA levels were negatively associated with production of interferon gamma (IFNɣ) in ex vivo lymphocytes after stimulation with tetanus and diphtheria toxoid. This study showed no effect of PFOA on infections during the first year of life, but the obtained results confirmed negative associations between the PFOA level and parameters of the immune response, which were observed in other epidemiological studies. The results connected with PFASs, including PFOA immunotoxicity concerning a study of 99 Norwegian children at 3 years old, revealed that maternal serum concentration of PFOA was linked to depleted vaccine responses in children, and in particular toward rubella vaccine, as well as rises in cases of the common cold and gastroenteritis [135]. Similarly, the research of Mogensen et al. (2015) [140] showed that prenatal exposure to PFOA was particularly associated with the pre-booster antibody concentration at 5 years of age, and concomitant exposure was linked to response to the 5-year booster, while concentrations of antibody at 7 years depended mostly on the current exposure to PFOA level. The prospective study was also conducted in adolescents (516 subjects) to assess PFOA-associated alterations in antibody responses to childhood vaccines at 7 years of age. The results showed that diphtheria but not tetanus antibody concentration was depleted by 25% at an increased serum level of PFOA at the age of 13. In adults, a decrease in antibody response to the influenza A H3N2 virus were associated with increasing serum PFOA levels; however, there were no associations with two other strains of influenza virus (influenza A H1N1 and influenza B) [141]. An interesting study was conducted by Grandjean et al. (2020) [142], who used (from Danish biobanks) samples of plasma from 323 non-pregnant subjects (aged 30–70 years) with confirmed SARS-CoV-2 infection and determined concentrations of five PFASs, including PFOA and PFBA. They showed that only the increased level of PFBA in plasma was linked to an elevated risk of a more severe course of COVID-19. The authors concluded that it may have been due to accumulation of PFBA in the lungs.

In order to elucidate the immunotoxic effect of PFASs, the EFSA panel (2020) [42] proposed transactivation of several nuclear receptors, as observed from in vivo and in vitro studies, including peroxisome proliferator-activated receptors (PPARs), nuclear factor kappa B (NF-κB), constitutive activated receptor (CAR), nuclear factor erythroid 2-related factor 2 (Nrf2), pregnane X receptor (PXR) and retinoid X receptor (RXR). Although some of these nuclear receptors may have an indirect effect on immune health, the following sections were focused on the modulation of NF-κB and PPARs. This selection was made because of the interactions of NF-κB and PPARs with the immune system, while data on other nuclear receptors are less conclusive.

### 6.4. Epigenotoxicity

Epigenetic modifications play a considerable role in the regulation of various functions of organisms during their development. These modifications include methylation of DNA, modifications of histones, and small non-coding RNA expression of regulating genes [143].

Liu and Irudayaraj (2020) [144] studied epigenetic changes and inhibitory mechanisms of PFOA in human breast epithelial cells (MCF7). They observed that exposure to PFOA at 100 μM and 200 μM (24 h or 48 h) changed the mobility of DNA (cytosine-5)-methyltransferase 3A (DNMT3A) and caused inhibition of enzymatic activity in DNA methyl transferases (DNMTs), which resulted in global DNA demethylation. Based on the distribution profile of histone 3 lysine 9 tri-methylation (H3K9me3), which is considered to be a hallmark for constitutive organization of heterochromatin, the authors deduced that PFOA (200 μM and 400 μM) significantly altered the organization of heterochromatin, increasing its redistribution around the periphery of the nucleus. The authors of this study proposed a detailed explanation for their findings. They suggested that PFOA caused dissociation of DNMTs from the nucleosome, which resulted in the loss of its function to methylate CpG sites even in the presence of increased expression of DNMT3A mRNA, which led to a reduced level of global methylation of DNA. At this level, the global 5-methylcytosine (5mC) loss induced changes in chromatin packaging at the single nucleosome level. The obtained results showed that PFOA caused epigenetic changes in heterochromatin packaging by a direct effect on DNMTs, which may enhance susceptibility to diseases, such as cancer.

DNA methylation is one of the crucial epigenetic mechanisms that is mainly regulated by ten-eleven translocation (TET) methylcytosine dioxygenases and DNMTs controlling gene expression by adding or removing methyl groups on cytosine at specific Cytosine–Guanine (CpG) sites in those genes [145].

Wen et al. (2020) [146] showed that PFOA caused global epigenetic changes, particularly DNA methylation, in mouse liver (1 mg or 10 mg/kg/day; 10 days), and induced tissue-specific changes in RNA binding proteins affecting alternative splicing factors. Moreover, PFOA caused gene expression in the mammalian target of the rapamycin (mTOR) pathway and decreased phosphatase and tensing homolog (Pten) expression, which is a primary inhibitor of the mTOR pathway. In another study, it was observed that PFOA decreased the expression of DNA methyltransferases (Dnmt1, Dnmt3a, Dnmt3b) in CD1 mice (1–20 mg/kg/day; 10 days), in the colon and small intestine. Moreover, they observed that expression of ten-eleven translocation genes (*Tet2* and *Tet3*) was dysregulated in the small intestine, while in the colon, *Tets* remained unaffected. The tight junction genes Claudins (*Cldn*), Occludin (*Ocln*), and Tight Junction Protein (Tjp) were also strongly affected in the small intestine. The authors concluded that PFOA induces DNA methylation and changes gene expression that is crucial for maintaining the physical barrier of the intestine, with stronger effects observed in the small intestine in comparison to the colon. Similarly, PFOA has been shown to induce epigenetic changes in the kidney of CD-1 mice (1–5 mg/kg/day; 10 days). The authors noted that PFOA caused variable methylation yielding 879 differentially methylated regions. The mRNA expression showed a significant rise in Dnmt1 and depleted RAS protein activator like 1 (Rasal1) expression, which is an early indicator of fibroblast activation in kidney. They also observed a considerable increase in histone deacetylase (Hdac) 1, 3 and 4, which are critically altered in some renal diseases. Furthermore, a substantial increase in the mRNA expression level of beta tumor growth factor (TGF-β) and alpha smooth muscle actin (α-SMA) was noted. Finally, the Kyoto Encyclopedia of Genes and Genomes (KEGG) and Gene oncology (Go) enrichment pathways analysis of decreased representation bisulfite data also showed their involvement in renal fibrosis. The authors concluded that the obtained results suggest that epigenetic alterations in kidney trigger the expression of early markers of fibroblast activation.

An interesting study was conducted by Ahmad et al. (2021) [145], who assessed the accumulation of PFOA in lungs and studied alterations in mRNA expression of methylation of DNA regulator genes DNA methyltransferases (Dnmts), as well as ten-eleven translocation (*Tets*) along with membrane proteins angiotensin transforming enzyme 2 (Ace2) and transmembrane Serine Protease 2 (*Tmprss2*) genes implicated in the infection of SARS-CoV-2 virus. They used CD1 mice that were orally exposed to PFOA at 5 mg or 20 mg/kg/day for 10 days. The authors observed that PFOA accumulated in lungs in a dose-dependent manner. They also found that Dnmts and *Tets* were considerably downregulated, while the expression levels of angiotensin-converting enzyme 2 (Ace2) and transmembrane serine protease 2 (Tmprss2) were significantly increased. Moreover, they noted considerable hypomethylation in CpG islands in the promotor region of *Tmprss2*. In conclusion, they stated that PFOA increased the expression of tested membrane receptors, possibly causing higher susceptibility of SARS-CoV-2 infections. The confirmation of the above findings may be the results of Grandjean et al. (2020) [142], who studied samples from 323 subjects (30–70 years old) from Denmark with known SARS-CoV-2 infection on the presence of PFASs. As a result, they showed that increased PFBA plasma concentrations (PFAS that accumulates in the lungs) were associated with an elevated risk of a more severe course of COVID-19.

### 6.5. Genotoxicity and Cancerogenicity

Genotoxicity refers to the capability of a substance of damaging the genetic material of a cell, leading to mutations that may finally cause cancer or hereditary genetic disorders. The substances that reveal a genotoxic influence can act directly or indirectly on DNA: for example, by ROS formation that causes oxidative deterioration of genetic material.

PFOA, PFHxA and PFBA have characteristics which are typical for the group of peroxisome proliferators. PPARa activation in in vivo models, including mice and rats, is believed to be connected to ROS induction and damage to DNA that may be implicated in peroxisome proliferator-mediated carcinogenesis. Nevertheless, this mode of activity has been shown to be highly species-specific and most probably has small importance in humans [147,148].

Eriksen et al. (2010) [149] studied the effect of perfluorinated compounds (PFCs), including PFOA and PFHxA, on intracellular ROS formation and DNA damage in HepG2 cells. The authors of this study showed that PFOA increased the ROS level by 1.52-fold, but did not cause damage to DNA, which was measured by analysis of DNA strand-breaks, alkali-labile sites and purine oxidation. They also noticed that PFHxA did not increase the ROS level nor cause DNA damage. Summing up, the achieved results proved that only PFOA was capable of inducing the ROS level, while both studied compounds did not exhibit genotoxic potential in a cell line representing the human liver. In an in vivo study, Crebelli et al. (2019) [150] evaluated the genotoxic potential of PFOA (0.1, 1 and 5 mg/kg body weight) and PFBA (5 mg/kg body weight) that were given in drinking water for mice for 5 weeks. They showed that PFOA administration at its highest dose caused considerable liver hypertrophy with signs of cell injury. The authors also observed that ALT and AST levels were increased, while no lipid peroxidation and oxidative stress occurred. After PFBA administration, only mild liver hypertrophy was noticed. Moreover, tested substances did not induce genotoxic alterations. The authors concluded that PFOA caused significantly stronger liver damage than PFBA, which was not due to oxidative stress, whereas none of the tested compounds induced genotoxic effects in liver or the other tested tissues, such as bone marrow (micronucleated reticulocytes), spleen (splenocytes) and testis.

According to the scientific literature focused on the study and analysis of carcinogenic substances, several features of PFASs, such as PFOA, can be recognized that assist in carcinogenic risk or the likelihood of cancer development. These characteristics embrace: metabolic activation, induction of oxidative stress, inflammation, immunosuppressive influence, and the ability to transform cells, making them “immortal” [148,151].

Among PFCAs, PFOA has been the most thoroughly investigated. It has been shown that exposure to PFOA is positively correlated with development of liver, pancreas, colon, breast, and testicle cancers [152]. The literature analysis has proven that PFOA is carcinogenic in mice [153], and large doses of PFOA induced hepatocellular adenomas in rats [95]. The mechanism of PFOA action connected to potential tumorigenic activity in breast cancer was studied by Pierozan et al. (2018) [154]. They used human breast epithelial cells (MCF-10A) that, after exposure to PFOA at 50 µM and 100 µM, showed a higher rate of proliferation by accelerating G0/G1 to an S phase transition of the cell cycle. Moreover, they observed that PFOA increased levels of cyclin-dependent kinases, including D1, CDK4 and CDK6, along with a depletion of the level of kinase inhibitor p27. It is worth noting that the estrogen receptor antagonist did not show any effect on promotion of cell proliferation by PFOA, whereas the PPARα antagonist was capable of preventing the proliferation of tested cells. The obtained results showed that the underlying mechanisms of action of PFOA implicated PPARα-dependent pathways. Interestingly, the authors noted that PFOA was able to stimulate cell migration and invasion, which may suggest its potential to induce neoplastic transformation of human breast epithelial cells.

In epidemiological studies, it has been proven that elevated serum concentrations of PFOA were associated with a higher risk of various cancer types [155].

Barry et al. (2013) [156] studied the correlation between residents in Mid-Ohio Valley (Washington, WV, USA) who were exposed to PFOA in drinking water polluted with chemical plant and cancer incidence. The cohort consisted of adults (n = 32,254) who were interviewed in 2008–2011 to obtain their medical history, whereas retrospective PFOA serum levels were determined for each participant from 1952 to 2011. The authors evaluated the correlation between cumulative PFOA exposure and 21 different cancer types, and found positive associations for kidney, thyroid and testicular cancers. In another epidemiological study conducted by Girardi and Merler (2019) [157], an association between PFASs exposure and mortality (1970–2018) of 462 male employees who had worked in the production of PFOA since 1968 was evaluated. They determined very high PFOA concentrations in the serum of 120 workers (2000–2013), which were from 19 to 91,900 ng/mL. They showed associations between cumulative PFOA levels and increased mortality related to liver cancer, as well as the malignant neoplasm of lymphatic and hematopoietic tissue. Studies concerning humans who were highly exposed to PFOA showed an association between exposure to this chemical and elevated incidence of kidney cancer, whereas it was unclear if PFOA or other PFASs behave as renal carcinogens, and thus, may increase the risk of renal cell carcinoma (RCC) in humans who are environmentally exposed. In another study, Shearer et al. (2021) [158] evaluated an association between the concentrations of PFOA in the serum of the general population of the U.S (with 324 RCC cases) and incidence of RCC risk. The experiment showed that there was a positive association between exposure to PFOA and RCC risk. The authors of this study concluded that PFOA is a renal carcinogen and may pose a significant threat to people [157]. A recent study that used serum samples from individuals occupationally exposed to PFOA, such as workers in chemical plants producing PFASs, as well as residents living near such facilities, showed a correlation between elevated PFOA levels and the incidence of breast cancer in women, particularly tumors lacking hormone receptors [159].

Since PFOA was previously classified as “possibly carcinogenic to humans,” many new studies have evaluated the relationship between exposure to this substance and cancer risk in both animals and humans.

In the light of these findings, in 2024, the International Agency for Research on Cancer (IARC) decided to reclassify PFOA as carcinogenic to humans (Group 1), taking into consideration sufficient evidence obtained from both human and animal studies, as well as mechanistic data. A panel of 30 scientists reached this conclusion based on:

- Sufficient evidence in animals, showing elevated incidences of benign and malignant tumors in both sexes;
- Strong evidence in humans, showing that PFOA causes epigenetic alterations (e.g., changes in methylation of CpG, modifications of histone and miRNA expression) and oxidative stress induction (e.g., elevated ROS formation);
- Immunosuppressive effects, including depletion of immune responses to vaccines and elevated susceptibility to infectious diseases, as PFOA has been observed to suppress production of cytokine and decrease lymphocyte levels [160].

The IARC expert group also point out that PFOA elevated cellular proliferation by promotion of the activity of the glycolytic pathway, and caused cell proliferation and migration. They also underlined that PFOA was able to influence pathways connected to the nutrient and energy supply, which can lead to cell death [153].

As for PFBA, the available data are insufficient to assess its carcinogenic potential. Animal studies have not shown satisfactory evidence concerning cancer induction by this substance [161]. For example, a 2-year toxicology and carcinogenicity study was conducted in male and female Sprague–Dawley rats, which were orally treated with PFBA (7 days per week) at doses ranging from 2.5 to 100 mg/kg/day (males) and from 5 to 200 mg/kg/day (females) [162]. As a result, there was no evidence that PFBA is able to induce tumors. Regarding PFHxA, no carcinogenic effects have been noticed in rats [162].

It may also be supposed that the dysregulation of cell signaling mechanisms by discussing compounds affecting gene expression and epigenetic toxicity, leads to abnormal cell growth in tissue, which results in growth and development pathologies, including inflammation, immunotoxicity and cancer.

The key molecular mechanisms underlying the toxicity of PFOA and its short-chain analogs are summarized in Table 8.

An overview of in vivo, ex vivo, in vitro, clinical and epidemiological studies on PFASs toxicity is presented in Table 9.

## 7. Conclusions

PFOA and its selected short-chain analogs—PFHxA and PFBA—constitutes a group of PFAAs. Due to their unique properties, i.e., stability, durability and lowering of surface tension, they are used in the industry, as well as in various everyday products. PFASs are named “perennial chemicals” because they exhibit resistance to degradation in the environment and show high accumulation in biota, including humans. It has been proven that the exposure of the general population to PFASs is mainly related to the consumption of contaminated food and drinking water, as well as dust inhalation. Those substances have been determined in surface water and, in higher concentrations, in ground waters, in which PFBA, PFHxA and PFOA have been found with higher frequency than other PFASs. Food, particularly fish, meat and meat products (PFOA) [143] and cereals (PFBA) [46], is the most significant source of human exposure to PFOA and its short-chain analogs. Dust adsorbing significant concentrations of PFBA, PFHxA and PFOA is also an essential source of the exposure of the human population to discussing toxicants. PFASs concentrations are not systematically measured in the air; however, the discussed compounds and PFBA in particular have been detected in the atmosphere.

PFOA has been produced for decades, but due to its toxicity, it is being gradually replaced by PFASs of shorter chains, including PFHxA and PFBA. Generally, PFASs, particularly PFOA and PFHxA, accumulate mainly in the liver, therefore inducing hepatoxicity. Nevertheless, PFBA accumulates more effectively in lungs in comparison to PFHxA and PFOA.

Several studies have shown deleterious effects of discussed PFASs on liver function, metabolism and morphological changes. The activation of PPARα by PFASs, including PFOA, PFHxS and PFBS, has been recognized as the primary mechanism of action in rodent hepatocyte-induced proliferation. It has also been observed that PFOA causes activation of p53 and blocks androgen receptor and nuclear receptor subfamily 1 group D member 1, which shows that a new adverse human-relevant molecular mechanism of PFASs exists, including its previously unknown effect on circadian rhythm. PFOA is also responsible for the gut microbiota dysbiosis, which is related to liver dysfunction. Moreover, occupational surveys have found associations between human exposure to PFBA, PFHxA and PFOA and higher risk of liver damage and its disfunction. Some PFASs have been recognized as endocrine-disrupting compounds. PFHxA have not induced alterations in endocrine system in various animal models. In contrast, PFOA induces endocrine disruption mainly by altering the functions of the ER. The authors suggested that the exposure to PFOA can impair reproductive function, possibly by altering CPY11A1 and testosterone levels. Endocrine-disrupting potential of PFOA has also been observed in occupational surveys, which show that it increases thyroid-stimulating hormone and T3 levels. Generally, the obtained results may point out that high concentrations of PFOA in serum are potentially associated with thyroid disease. The studies have also shown that PFBA negatively influences TH levels, while for PFOA and PFHxA, such a correlation has not been found. Immunotoxic potential of PFASs has been shown both in vitro and in vivo, as well as in epidemiological studies. In mice, PFOA caused suppression of antigen-specific IgM antibody production, splenic and thymic atrophy, and altered T-cell phenotypic distribution. PFOA also altered Th1 cytokine balance and decreased pro-inflammatory cytokines. It has been concluded that PFOA may be recognized as an immunosuppressant in rodents. Several epidemiological studies have shown associations between PFOA serum levels and diagnosis of asthma in children and adults, as well as development of osteoarthritis, ulcerative colitis and rheumatoid arthritis in adults. Moreover, the maternal serum concentration of PFOA was linked to depleted various vaccine responses in children. Interestingly, increased PFBA, but not the PFOA level, was positively associated with elevated risk of a more severe course of COVID-19, which might be due to higher accumulation of PFBA in lungs. The EFSA panel suggested that transactivation of several nuclear receptors, particularly PARPs and NF-κB, may be responsible for the immunotoxic potential of PFASs, such as PFOA and PFBA. The studies have also shown that PFOA exhibits epigenetic potential, as it induces global DNA demethylation and significantly alters the organization of heterochromatin, which may contribute to the development of various diseases, such as cancer. It was also observed that PFOA caused DNA global methylation in liver and changed gene expression that is crucial for maintaining the physical barrier of the intestine [166]. PFOA also induced a considerable increase in histone deacetylases, which are critically important in some renal diseases. Epigenetic changes induced by PFOA seem to be the most probably involved in development of various cancer types. The results concerning epigenetic activity of PFHxA and PFBA are scarce. It was noticed that increased PFBA plasma concentration was associated with elevated risk of a severe course of COVID-19. PFOA, PFHxA and PFBA have shown capabilities which are characteristic for the group of peroxisome proliferators that are recognized as non-genotoxic chemical substances. The results of the studies have shown that although PFOA, PFHxA and PFBA induced ROS formation, they did not cause damage to DNA. Among PFCAs, the carcinogenic potential of PFOA has been the most thoroughly studied. It has been proven that exposure of rodents to PFOA is positively associated with cancers of the liver, pancreas, colon, breast, and testicles. Obtained results have shown that the underlying mechanisms of carcinogenic PFOA action involves PPARα-dependent and kinases-dependent pathways. Similarly, in epidemiological studies, it has been proven that increased serum PFOA concentrations have been associated with a higher risk of different cancer types, including liver, kidney, thyroid and testicular cancers, as well as malignant neoplasm of lymphatic and hematopoietic tissue. In the light of these findings, in 2024, the IARC classified PFOA as carcinogenic to humans (Group 1). As for PFBA, the available data are insufficient to evaluate its carcinogenic potential—animal studies have not provided conclusive evidence of its carcinogenicity. Regarding PFHxA, no carcinogenic effects have been observed in rats.

It should be emphasized that there is a limited number of toxicological studies in vitro and in vivo, as well as epidemiological surveys regarding action of PFHxA and PFBA. Considering that these substances prefer bioaccumulation in different organs (PFHxA accumulates mainly in liver, while PFBA accumulates mostly in lungs and kidneys), the assessment of the toxic effects of these substances on humans, which currently poses the biggest challenge, is even more difficult.

## Figures and Tables

**Table 1:** Chemical structures of PFOA, PFHxA and PFBA.

PFOA	PFHxA	PFBA
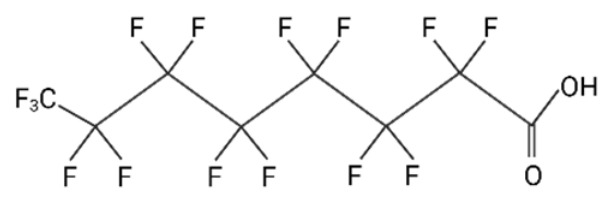	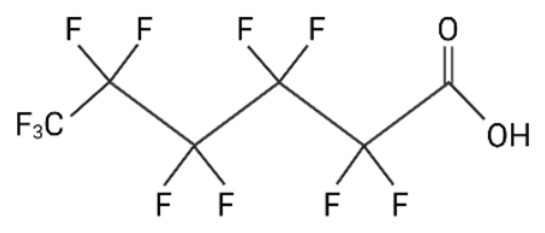	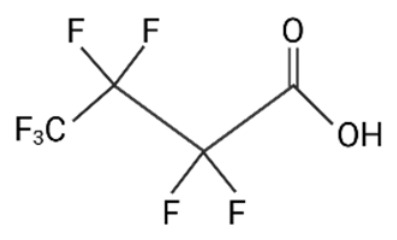

**Table 2:** Physiochemical properties of PFOA, PFHxA and PFBA.

Property	PFOA	PFHxA	PFBA	Reference
CAS number	335-67-1	307-24-4	375-22-4	[12,13,14]
molecular formula	C_7_F_15_COOH	C_5_F_11_COOH	C_3_F_9_COOH	[12,13,14]
molecular mass [g/mol]	414.07	314	214	[12,13,14]
physical state at room temperature and atmospheric pressure	white/cream solid	colorless liquid	colorless liquid	[3,12,13,14,15]
boiling point [°C]	188 °C	157 °C	121 °C	[13,14,16]
melting point [°C]	54.3 °C	14.0 °C	−17.5 °C	[13,14,16]
density [g/cm^3^]	1.79 g/cm^3^	1.69 g/cm^3^ *	1.65 g/cm^3^	[12,13,14,17]
vapor pressure [mm Hg] at 25 °C	0.525	0.908	15.8 *	[12,13,14]
pKa	2.5	−0.16	0.08 *	[12,13,14,18]
Log P	2.69	2.51	1.43	[12,13,14]
water solubility [mg/L] at 25 °C	9.5 × 10^3^	1.57 × 10^4^	2.14 × 10^5^	[14]

Abbreviations: CAS—Chemical Abstracts Service number, g/mol—grams per mole; mg/L—milligrams per liter; °C—Celsius degree; mm Hg—millimeters of mercury, * *p*-value.

**Table 3:** Presence of PFOA, PFHxA and PFBA in the environment.

Compound	Groundwater (ng/L)	Surface Water (ng/L)	Air (pg/m^3^)	References
PFOA	ND–4470 (Sweden) ND–2510 (China) ND–34.96 (Thailand) ND–3260 (Canada) ND–24,000 (USA)	ND–522 (Sweden) ND–223.8 (China) ND–10.7 (Thailand) 1.6–73 (Canada) 11,000 (USA) 1.0–7.5 (Bangladesh) 4.0–8.0 (Brazil) ND–13 (Australia)	1.25–64.28	[28,30,31,32,33,34,35,36]
PFHxA	ND–4000 (USA) ND–90 (Italy)	1.8–8.1 (Bangladesh) 0.4–173 (Canada) 1.0–3.0 (Brazil) 8.04–47.3 (China)	0.3–1.2	[34,35,36,37,38]
PFBA	ND–70 (Italy)	1.6–15 (Bangladesh) 1.6–73 (Canada) 0.2–2 (Brazil) 6.35–53.5 (China) ND–5.7 (Australia)	0.1–0.7	[34,35,36,38,39]

ND—not detected.

**Table 4:** Presence of PFOA, PFHxA and PFBA in human surroundings.

Compound	Food (ng/g)	Drinking Water (ng/L)	Dust (ng/g)	References
PFOA	perch 5.22–67.8 eels 5.73–68.8 mussels 8.56–157 eggs 0.05–4.83 meat 8.78–12.1 pork offal 2.15–283 sea food 2.33–54.6 onion 65–825 celery 900–2678 carrots 1000–5303	100 (Sweden) 5.95–19.3 (China) 7.6 (Canada) 4300 (USA) 10.5 (China)	10–653	[28,37,39,40,41,42,43,44,45,46]
PFHxA	beef 1.21 chicken 1.02 fish 1.92–41.1 sea food ND–3.77 oysters 1.2 pork offal ND–5.14 eggs 0.82	1400 (USA) 2.7 (China)	3.6–72.5	[39,40,41,44,45]
PFBA	eggs 81.4 meat 0.06–3.0 pork offal ND–20 crustaceans ND–2.0 cereals max. 140	1.3 (Spain) 17.87 (China) 6.9–9.0 (USA) 8.84 (China)	271 pg/m^3^	[41,42,43,45,47,48]

ND—not detected.

**Table 5:** Comparison of permissible levels of PFOA in selected food products. Own elaboration based on the European Union Commission Regulation [48].

Food Products	Maximum Permissible Level of PFOA (µg/kg Dry Weight)
eggs	0.3
fish meat—Baltic herring, sprat, pike, catfish, tench, whitefish, wild salmon, wild trout	1.0
crustaceans and mussels	0.7
sheep meat	0.2
pork, beef, poultry and mutton offal	0.7

**Table 6:** (a) Presence of PFOA, PFHxA, and PFBA in the environmentally exposed population. (b) Presence of PFOA, PFHxA, and PFBA in the occupationally exposed population.

Compound	Material	Concentration (ng/mL)	Subjects	Location	Year of Study	Literature
(a)						
PFOA	serum	0.3–38.5	men, women	Sweden	2008–2020	[56]
PFHxA	serum	0.1–100	men, women	Norway	2013–2014	[61]
PFBA	serum	0.008–2.5	men, women	USA	2020	[64]
(b)						
PFOA	serum	2.9–11	men, women, breastfeeding women	Sweden	2018	[65]
	full of blood	4.8–535	men	Sweden	2007–2010	[60]
PFHxA	serum	0.07–12.2	men	Sweden	2007–2009	[62]
PFBA	serum	3.7–5.4	men	Norway	2008–2009	[66]

**Table 7:** The concentrations of PFOA, PFHxA, and PFBA in urine, human milk, serum and solid tissues.

Compound	Material	Concentration	Subjects	Location	Year of Study	Literature
PFOA	urine	7.3 ng/mL	men, women	China	2019	[57]
	human milk	336 ng/L	breastfeeding women	China	2020–2021	[58]
	hair	0.1–6 ng/g	men, women	Spain	2010–2011	[59]
	liver	4 ng/g	men, women	Spain	2008	[59]
	bone	20.9 ng/g	men, women	Spain	2008	[59]
	lung	12.1 ng/g	men, women	Spain	2008	[59]
	kidney	1.5 ng/g	men, women	Spain	2008	[59]
PFHxA	urine	0.3 ng/mL	men, women, children from 7 years of age	USA	2013–2014	[67]
	brain	141 ng/g	men, women	Spain	2008	[59]
	liver	68.3 ng/g	men, women	Spain	2008	[59]
	bone	1.5 ng/g	men, women	Spain	2008	[59]
	lung	207 ng/g	men, women	Spain	2008	[59]
	kidney	2.7 ng/g	men, women	Spain	2008	[59]
PFBA	urine	0.1–0.9 ng/mL	men, women	Spain	2010–2011	[59]
	brain	1.4 ng/g	men, women	Spain	2008	[59]
	liver	3 ng/g	men, women	Spain	2008	[59]
	bone	0.8 ng/g	men, women	Spain	2008	[59]
	lung	807 ng/g	men, women	Spain	2008	[59]
	kidney	263 ng/g	men, women	Spain	2008	[59]

**Table 8:** Summary of molecular mechanisms of action of PFOA and its selected short-chain analogs (PFHxA, PFBA) highlighting both shared and compound-specific pathways based on selected studies discussed in Section 6.

Mechanism	PFOA	PFHxA	PFBA	Molecular Targets/Pathway	References
PPARα activation	Strong activation	Strong activation	Strong activation	PPARα, lipid metabolism genes, β-oxidation	[84,87,93]
Oxidative stress (ROS/RNS generation)	Strong induction	Strong induction	Strong induction	ROS, antioxidant enzymes (SOD, GSH), mitochondrial dysfunction	[79,94,122]
Endocrine disruption	Well-documented (ER activation, steroidogenesis disruption)	Weak or negligible	Limited evidence	Estrogen receptor (ER), androgen pathways, thyroid hormones	[106,110,111,112]
Immunotoxicity	Well-documented (reduced antibody response, cytokine modulation)	Weak to moderate	Moderately supported	Cytokines (IL-6, TNF-α), T/B cells, IgM response	[123,125,135]
Epigenetic modifications	Well-documented (DNA methylation, DNMT inhibition, histone changes)	Poorly studied	Limited evidence	DNMTs, TET enzymes, histone modifications (H3K9me3)	[144,145,146]
Lipid metabolism disruption	Well-documented	Moderately supported	Limited evidence	PPARα, lipid transport, cholesterol homeostasis	[88,91,92]
Mitochondrial dysfunction/energy metabolism	Well-documented	Limited evidence	Moderately supported	Depleted ATP production, mitochondrial respiration	[122]
Inflammatory pathways activation	Well-documented	Limited evidence	Moderately supported	NF-κB, cytokines, immune signaling	[42,98]
Carcinogenic-related pathways	Well-documented (non-genotoxic mechanisms)	Not well-established	Not established	Cell proliferation, ERK/MAPK, PPARα	[148,154,156]

**Table 9:** Overview of selected in vivo, ex vivo, in vitro, clinical and epidemiological studies on PFASs toxicity.

	Study Type	Sample/Model	Sample Size	Dose/Exposure	Conclusions	References
**Hepatotoxicity**	In vivo	Sprague–Dawley rats	10 rats/sex/group	Up to 200 mg/kg/day PFHxA by gavage for 90 days	PFHxA exposure was associated with lower red blood cell parameters, higher reticulocyte counts and lower globulin level, higher liver enzyme activities, centrilobular hepatocellular hypertrophy	[81]
	In vivo	Pregnant Kunming mice and female offspring	50 pregnant mice (5 groups of 10)	0, 1, 2.5, 5, 10 mg/kg/day PFOA (gestational days 0–17)	Prenatal PFOA exposure impairs pup growth and development, induces liver damage, disrupts PPAR-α-mediated fatty acid oxidation, causes oxidative stress, and reduces histone acetylation	[86]
	Ex vivo	FRG liver-chimeric humanized mice (human hepatocytes)	n=3 for each experiment	0.067 mg/L PFOA, 0.145 mg/L PFOS, 1 mg/L GenX in drinking water for 28 days	PFOS decreased total cholesterol and LDL/VLDL, GenX increased LDL/VLDL, and PFOA had no significant effect	[87]
	Clinical	Children with nonalcoholic fatty liver disease (NAFLD)	74	Serum PFASs concentrations (median): PFOA 3.42 ng/mL, PFOS 3.59 ng/mL, and PFHxS 1.53 ng/mL	Higher PFOS and PFHxS levels were linked to increased risk of nonalcoholic steatohepatitis (NASH) and liver fibrosis predominantly in obese children, with PFHxS also associated with inflammation and higher NAFLD activity. Elevated PFASs exposure correlated with distinct metabolic alterations.	[102]
	Epidemiology	US adult participants (NHANES)	9523	Environmental exposure measured in serum (geometric mean): PFOA-nonobese: 2.2 ng/mL, obese: 2.0 ng/mL, PFOS-nonobese: 6.3 ng/mL, obese: 5.5 ng/mL, PFDA–nonobese: 0.21 ng/mL, obese: 0.18ng/mL	In obese participants, ALT was positively associated with PFOA, PFHxS, and PFNA serum concentrations, while PFOA and PFNA were also linked to increased gamma-glutamyl transpherase (GGT)	[101]
**Immunotoxicity**	In vivo	Mice	A total of 40 mice per endpoint	PFOA in drinking water: 0–30 mg/kg/day	PFOA exposure suppressed IgM antibody production	[125]
	Prospective cohort	Infants/children cohort (Norway)	99 mother-child pairs	Maternal serum level (geometric mean) of PFOA: 1.1 ng/mL, PFOS: 5.6 ng/mL	Prenatal PFASs exposure was linked to immunosuppression in early childhood, evidenced by lower anti-rubella antibody levels and increased episodes of common cold and gastroenteritis	[135]
	Ex vivo	1-year-old children	101	PFOA plasma level (geometric mean): 3.8 ng/mL	PFOA levels were inversely associated with IFNɣ production in stimulated lymphocytes following tetanus and diphtheria toxoid exposure	[139]
	In vitro	Human Namalwa B lymphocyte and human Jurkat T lymphocyte cells	cell culture experiments	Concentrations up to 100 µM of PFASs (PFOA, PFBA, PFNA, PFDA and others)	PFASs reduce RAG1 and RAG2 expression and suppress IL-2 promoter activity, potentially impairing antibody responses	[163]
**Cancerogenity**	In vitro	Human mammary epithelial cell line MCF-10A	cell culture experiments	50–1000 µM of PFOA	PFOA promotes migration and invasion of human breast epithelial cells, indicating its potential to induce neoplastic transformation	[154]
	Epidemiology (C8 cohort)	Residents PFOA in contaminated water areas (C8 cohort cancer study), adults	32,254	PFOA-contaminated drinking water exposures (median): community 24.2 ng/mL, workers 112.7 ng/mL	PFOA exposure was associated with an increased risk of kidney and testicular cancer; however, interpretation is limited by the survivor cohort design and the high lethality of certain malignancies, such as pancreatic and lung cancer	[156]
	Epidemiology (occupational cohort)	Factory workers exposed occupationally	462 males	Occupational exposure to PFOA (geometric mean): 4048 ng/mL	Workers with the highest cumulative PFOA exposure had a significantly increased risk of liver cancer, liver cirrhosis, diabetes, and hematologic malignancies	[157]
	Epidemiology (case–control of renal carcinoma, RCC)	Renal cell carcinoma cases and controls	324 cases	Serum PFOA measurements (geometric mean): 3.6–4.8 ng/mL (depending on the study group)	A statistically significant positive relationship was observed between prediagnostic serum PFOA levels and subsequent RCC risk	[158]
**Reproductive disorder**	In vivo	Male mice	80 (5 groups of 16)	0–20 mg/kg/day PFOA by gavage for 28 days	PFOA impaired seminiferous tubules, decreased testicular testosterone and progesterone in a dose-dependent manner, and reduced sperm quality	[112]
	In vivo	CD-1 mice, gestational exposure studies	50 dams	Various gestational exposure regimes (i.e., 0, 1, or 5 mg PFOA/kg/day)	Gestational and chronic low-dose PFOA exposure impaired mammary gland development and lactational differentiation across generations in mice	[113]
**Epigenotoxicity**	In vitro	Human breast epithelial cells (MCF7)	cell culture experiments	100 μM and 200 μM (24 h or 48 h)	PFOA altered the mobility of DNA (cytosine-5)-methyltransferase 3A (DNMT3A) and inhibited DNA methyltransferase (DNMT) activity, leading to global DNA demethylation	[144]
**Endocrine disruption**	In vivo	Cynomolgus monkeys (male and female)	6–8 monkeys/sex/dose	0, 0.03, 0.15, 0.75 mg/kg/day orally for 182 days	Adverse effects were observed at 0.75 mg/kg/day (PFOS) and included mortality in 2 of 6 males, decreased body weight, increased liver weight, and reduced levels of cholesterol, triiodothyronine (without hypothyroidism), and estradiol.	[76]
	In vitro	Human osteosarcoma Saos-2 cells and epithelial colorectal adenocarcinoma Caco-2 cells	cell culture experiments	PFOA 400 ng/mL	PFOA disrupts vitamin D receptor signaling through endocrine interference, altering gene responses and reducing osteoblast mineralization	[164]
	In vitro	Primary bovine granulosa cells	cell culture experiments	0, 4, and 40 μM PFOA for 48 and 96 h	PFOA may impair granulosa cell steroidogenesis by inducing mitochondrial dysfunction	[165]

## Data Availability

No new data were created or analyzed in this study. Data sharing is not applicable to this article.

## Abbreviations

The following abbreviations are used in this manuscript:

17OHP	17-hydroxyprogesterone
17β-HSD	17β-hydroxysteroid dehydrogenase
A	Androgen
Ace2	Membrane proteins angiotensin transforming enzyme 2
AFFF	Aqueous film-forming foams
ALT	Alanine aminotransferase
ATSDR	Agency for Toxic Substances and Disease Registry
BKMR	Bayesian Kernel Machine Regression
CAR	Constitutive activated receptor
CERI	Centre for Educational Research and Innovation
Cldn	Genes Claudins
CpG	Cytosine at specific Cytosine–Guanine sites
CYP11A1	Cytochrome P450 cholesterol side-chain cleavage enzyme
Cyp7a1	Cholesterol 7 alpha-hydroxylase
DNA	Deoxyribonucleic acid
DNMT3A	DNA (cytosine-5)-methyltransferase 3A
DNMTs	DNA methyl transferases
Dnmts	DNA regulator genes DNA methyltransferases
EFSA	European Food Safety Authority
ER	Estrogen receptor
ERK	Extracellular receptor kinase
ERα	Estrogen receptor α
FT3	Free triiodothyronine
FT4	Free thyroxine
GJIC	Gap-junctional intercellular communication
GSH	Glutathione
H	Estrogen
H3K9me3	Histone 3 lysine 9 tri-methylation
Hdac	Histone deacetylase
Hmgcr	3-hydroxy-3-methylglutaryl-CoA reductase
hPPARα	Human peroxisome proliferator receptor alpha
IARC	International Agency for Research on Cancer
IFNɣ	Interferon gamma
IgM	Immunoglobulin M
INSL3	Insulin like-factor 3
Ldlr	Low-density lipoprotein receptor
MAPK	Mitogen-activated protein kinase
MCF7	Human breast epithelial cells
mRNA	Messenger RNA
mTOR	Mammalian target of rapamycin
NALFD	Nonalcoholic fatty liver disease
NASH	Nonalcoholic steatohepatitis
NF-κB	Nuclear factor kappa B
NHANES	National Health and Nutrition Examination Survey
NR1D1	Nuclear receptor subfamily 1 group D member 1
Nrf2	Nuclear factor erythroid 2-related factor 2
Ocln	Genes occlusion
PBMCs	Human peripheral blood mononuclears cells
PC-PLC	Phosphatidylcholine-specific phospholipase C
PFAAs	Perfluoroalkyl acids
PFASs	Polyfluoroalkyl substances
PFBA	Perfluorobutanoic acid
PFCs	Perfluorinated compounds
PFHxA	Perfluorohexanoic acid
PFOA	Perfluorooctanoic acid
PFPrA	Perfluoropropanoic acid
PFTrDA	Perfluorotriodecanoic acid
PFUnDA	Perfluoroundecanoic acid
PPARα PLC	Peroxisome proliferator receptor alpha Polymers of low concern
Pten PTFE	Phosphatase and tensing homolog Polytetrafluoroethylene
PXR	Pregnane X receptor
Rasal1	RAS protein activator like 1
RCC	Renal cell carcinoma
RNA	Ribonucleic acid
ROS	Reactive oxygen species
RXR	Retinoid X receptor
SOD	Superoxide dismutase
sRBCs	Sheep red blood cells
T	Thyroid
T3	Triiodothyronine
T4	Thyroxin
TDAR	T-Dependent Antibody Response assay
TDI	Tolerable daily intake
TET	Ten-eleven translocation
TGF-β	Beta tumor growth factor
TGN	Thyroglobulin
Th	T helper cells
THs	Thyroid hormones
Tjp	Genes Tight Junction Protein
Tmprss2	Transmembrane Serine Protease 2
Treg	Regulatory T-cell
TSH	Thyroid stimulating hormone
TSI	Thyroid-stimulating immunoglobulin
TT3	Total triiodothyronine
TT4	Total thyroxine
US EPA	US Environmental Protection Agency
WHO	World Health Organization
α-SMA	Alpha smooth muscle actin

## References

[1] Groh K.J., Geueke B., Martin O., Maffini M., Muncke J. (2021). Overview of Intentionally Used Food Contact Chemicals and Their Hazards. Environ. Int..

[2] Ehrlich V., Bil W., Vandebriel R., Granum B., Luijten M., Lindeman B., Grandjean P., Kaiser A.-M., Hauzenberger I., Hartmann C. (2023). Consideration of Pathways for Immunotoxicity of Per- and Polyfluoroalkyl Substances (PFAS). Environ. Health.

[3] Spyrakis F., Dragani T.A. (2023). The EU’s Per- and Polyfluoroalkyl Substances (PFAS) Ban: A Case of Policy over Science. Toxics.

[4] Hofmann A., Mishra J.S., Yadav P., Dangudubiyyam S.V., Blesson C.S., Kumar S. (2023). PFOS Impairs Mitochondrial Biogenesis and Dynamics and Reduces Oxygen Consumption in Human Trophoblasts. J. Environ. Sci. Public Health.

[5] India-Aldana S., Yao M., Midya V., Colicino E., Chatzi L., Chu J., Gennings C., Jones D.P., Loos R.J.F., Setiawan V.W. (2023). PFAS Exposures and the Human Metabolome: A Systematic Review of Epidemiological Studies. Curr. Pollut. Rep..

[6] Sunderland E.M., Hu X.C., Dassuncao C., Tokranov A.K., Wagner C.C., Allen J.G. (2019). A Review of the Pathways of Human Exposure to Poly- and Perfluoroalkyl Substances (PFASs) and Present Understanding of Health Effects. J. Expo. Sci. Environ. Epidemiol..

[7] Jian J.-M., Chen D., Han F.-J., Guo Y., Zeng L., Lu X., Wang F. (2018). A Short Review on Human Exposure to and Tissue Distribution of Per- and Polyfluoroalkyl Substances (PFASs). Sci. Total Environ..

[8] D’Hollander W., De Voogt P., De Coen W., Bervoets L., De Voogt P. (2010). Perfluorinated Substances in Human Food and Other Sources of Human Exposure. Reviews of Environmental Contamination and Toxicology.

[9] Podder A., Sadmani A.H.M.A., Reinhart D., Chang N.-B., Goel R. (2021). Per and Poly-Fluoroalkyl Substances (PFAS) as a Contaminant of Emerging Concern in Surface Water: A Transboundary Review of Their Occurrences and Toxicity Effects. J. Hazard. Mater..

[10] Danish Environmental Protection Agency (2015). Alternatives to Perfluoroalkyl and Polyfluoro-Alkyl Substances (PFAS) in Textiles; LOUS Survey of Chemical Substances in Consumer Products.

[11] Hull S.D., Deen L., Petersen K.U., Jensen T.K., Hammer P., Wils R.S., Frankel H.N., Ostrowski S.R., Tøttenborg S.S. (2023). Time Trends in Per- and Polyfluoroalkyl Substances (PFAS) Concentrations in the Danish Population: A Review Based on Published and Newly Analyzed Data. Environ. Res..

[12] National Library of Medicine Perfluorohexanoic Acid. https://pubchem.ncbi.nlm.nih.gov/compound/Perfluorohexanoic-Acid#section=Toxicity.

[13] US Environmental Protection Agency CompTox Chemicals Dashboard. https://comptox.epa.gov/dashboard.

[14] Agency for Toxic Substances and Disease Registry (2018). Toxicological Profile for Perfluoroalkyls.

[15] Royal Society of Chemistry Perfluorohexanoic Acid (CAS 307-24-4). https://www.chemspider.com/Chemical-Structure.60864.html.

[16] Lide D.R. (2003). CRC Handbook of Chemistry and Physics.

[17] Savu P.M. (2000). Fluorinated Higher Carboxylic Acids. Kirk-Othmer Encyclopedia of Chemical Technology.

[18] Ylinen M., Kojo A., Hanhijärvi H., Peura P. (1990). Disposition of Perfluorooctanoic Acid in the Rat after Single and Subchronic Administration. Bull. Environ. Contam. Toxicol..

[19] Chen Y.-F., Liu T., Hu L.-X., Chen C.-E., Yang B., Ying G.-G. (2024). Unveiling Per- and Polyfluoroalkyl Substance Contamination in Chinese Paper Products and Assessing Their Exposure Risk. Environ. Int..

[20] Prevedouros K., Cousins I.T., Buck R.C., Korzeniowski S.H. (2006). Sources, Fate and Transport of Perfluorocarboxylates. Environ. Sci. Technol..

[21] Strynar M.J., Lindstrom A.B. (2008). Perfluorinated Compounds in House Dust from Ohio and North Carolina, USA. Environ. Sci. Technol..

[22] Deng Y., Liang Z., Lu X., Chen D., Li Z., Wang F. (2021). The Degradation Mechanisms of Perfluorooctanoic Acid (PFOA) and Perfluorooctane Sulfonic Acid (PFOS) by Different Chemical Methods: A Critical Review. Chemosphere.

[23] Gebbink W.A., Ullah S., Sandblom O., Berger U. (2013). Polyfluoroalkyl Phosphate Esters and Perfluoroalkyl Carboxylic Acids in Target Food Samples and Packaging--Method Development and Screening. Environ. Sci. Pollut. Res. Int..

[24] Kotthoff M., Müller J., Jürling H., Schlummer M., Fiedler D. (2015). Perfluoroalkyl and Polyfluoroalkyl Substances in Consumer Products. Environ. Sci. Pollut. Res. Int..

[25] Buck R.C., Franklin J., Berger U., Conder J.M., Cousins I.T., de Voogt P., Jensen A.A., Kannan K., Mabury S.A., van Leeuwen S.P.J. (2011). Perfluoroalkyl and Polyfluoroalkyl Substances in the Environment: Terminology, Classification, and Origins. Integr. Environ. Assess. Manag..

[26] Agency for Toxic Substances and Disease Registry (2015). Toxicological Profile for Perfluoroalkyls.

[27] Leung S.C.E., Shukla P., Chen D., Eftekhari E., An H., Zare F., Ghasemi N., Zhang D., Nguyen N.-T., Li Q. (2022). Emerging Technologies for PFOS/PFOA Degradation and Removal: A Review. Sci. Total Environ..

[28] Wee S.Y., Aris A.Z. (2023). Environmental Impacts, Exposure Pathways, and Health Effects of PFOA and PFOS. Ecotoxicol. Environ. Saf..

[29] Runkel A.A., Stajnko A., Snoj Tratnik J., Mazej D., Horvat M., Přibylová P., Kosjek T. (2023). Exposure of Children and Adolescents from Northeastern Slovenia to Per- and Polyfluoroalkyl Substances. Chemosphere.

[30] Wee S.Y., Aris A.Z. (2023). Revisiting the “Forever Chemicals”, PFOA and PFOS Exposure in Drinking Water. npj Clean Water.

[31] Anderson J.K., Luz A.L., Goodrum P., Durda J. (2019). Perfluorohexanoic Acid Toxicity, Part II: Application of Human Health Toxicity Value for Risk Characterization. Regul. Toxicol. Pharmacol..

[32] Randazzo A., Pavan F., Gea M., Maffiotti A. (2025). Perfluoroalkyl Substances (PFASs) in Groundwater and Surface Water in the Turin Metropolitan Area (Italy): An Attempt to Unravel Potential Point Sources and Compliance with Environmental/Drinking Water Quality Standards. Sci. Total Environ..

[33] Zhou J., Baumann K., Surratt J.D., Turpin B.J. (2022). Legacy and Emerging Airborne Per- and Polyfluoroalkyl Substances (PFAS) Collected on PM2.5 Filters in Close Proximity to a Fluoropolymer Manufacturing Facility. Environ. Sci. Process Impacts.

[34] Morales-McDevitt M.E., Dunn M., Habib A., Vojta S., Becanova J., Lohmann R. (2022). Poly- and Perfluorinated Alkyl Substances in Air and Water from Dhaka, Bangladesh. Environ. Toxicol. Chem..

[35] US Environmental Protection Agency (2022). Drinking Water Health Advisories for Perfluorooctanoic Acid (PFOA).

[36] Schweizer C., Edwards R.D., Bayer-Oglesby L., Gauderman W.J., Ilacqua V., Jantunen M.J., Lai H.K., Nieuwenhuijsen M., Künzli N. (2007). Indoor Time-Microenvironment-Activity Patterns in Seven Regions of Europe. J. Expo. Sci. Environ. Epidemiol..

[37] Lalonde B., Garron C. (2022). Perfluoroalkyl Substances (PFASs) in the Canadian Freshwater Environment. Arch. Environ. Contam. Toxicol..

[38] Björnsdotter M.K., Hartz W.F., Kallenborn R., Ericson Jogsten I., Humby J.D., Kärrman A., Yeung L.W.Y. (2021). Levels and Seasonal Trends of C1-C4 Perfluoroalkyl Acids and the Discovery of Trifluoromethane Sulfonic Acid in Surface Snow in the Arctic. Environ. Sci. Technol..

[39] Khair Biek S., Khudur L.S., Rigby L., Singh N., Askeland M., Ball A.S. (2024). Assessing the Impact of Immobilisation on the Bioavailability of PFAS to Plants in Contaminated Australian Soils. Environ. Sci. Pollut. Res. Int..

[40] Teunen L., Bervoets L., Belpaire C., De Jonge M., Groffen T. (2021). PFAS Accumulation in Indigenous and Translocated Aquatic Organisms from Belgium, with Translation to Human and Ecological Health Risk. Environ. Sci. Eur..

[41] Chen W.-L., Bai F.-Y., Chang Y.-C., Chen P.-C., Chen C.-Y. (2018). Concentrations of Perfluoroalkyl Substances in Foods and the Dietary Exposure among Taiwan General Population and Pregnant Women. J. Food Drug Anal..

[42] Schrenk D., Bignami M., Bodin L., Chipman J.K., Del Mazo J., Grasl-Kraupp B., Hogstrand C., Hoogenboom L.R., Leblanc J.-C., EFSA Panel on Contaminants in the Food Chain (EFSA CONTAM Panel) (2020). Risk to Human Health Related to the Presence of Perfluoroalkyl Substances in Food. EFSA J..

[43] Ramírez Carnero A., Lestido-Cardama A., Vazquez Loureiro P., Barbosa-Pereira L., Rodríguez Bernaldo de Quirós A., Sendón R. (2021). Presence of Perfluoroalkyl and Polyfluoroalkyl Substances (PFAS) in Food Contact Materials (FCM) and Its Migration to Food. Foods.

[44] Susmann H.P., Schaider L.A., Rodgers K.M., Rudel R.A. (2019). Dietary Habits Related to Food Packaging and Population Exposure to PFASs. Environ. Health Perspect..

[45] Weiss J.M., Jones B., Koekkoek J., Bignert A., Lamoree M.H. (2021). Per- and Polyfluoroalkyl Substances (PFASs) in Swedish Household Dust and Exposure of Pet Cats. Environ. Sci. Pollut. Res. Int..

[46] Yao Y., Zhao Y., Sun H., Chang S., Zhu L., Alder A.C., Kannan K. (2018). Per- and Polyfluoroalkyl Substances (PFASs) in Indoor Air and Dust from Homes and Various Microenvironments in China: Implications for Human Exposure. Environ. Sci. Technol..

[47] Wang W., Zhou W., Wu S., Liang F., Li Y., Zhang J., Cui L., Feng Y., Wang Y. (2019). Perfluoroalkyl Substances Exposure and Risk of Polycystic Ovarian Syndrome Related Infertility in Chinese Women. Environ. Pollut..

[48] European Commission (2022). Commission Regulation (EU) 2022/2388 of 7 December 2022.

[49] Davis K.L., Aucoin M.D., Larsen B.S., Kaiser M.A., Hartten A.S. (2007). Transport of Ammonium Perfluorooctanoate in Environmental Media near a Fluoropolymer Manufacturing Facility. Chemosphere.

[50] Stefano P.H.P., Roisenberg A., D’Anna Acayaba R., Roque A.P., Bandoria D.R., Soares A., Montagner C.C. (2023). Occurrence and Distribution of Per-and Polyfluoroalkyl Substances (PFAS) in Surface and Groundwaters in an Urbanized and Agricultural Area, Southern Brazil. Environ. Sci. Pollut. Res. Int..

[51] Zhao P., Xia X., Dong J., Xia N., Jiang X., Li Y., Zhu Y. (2016). Short- and Long-Chain Perfluoroalkyl Substances in the Water, Suspended Particulate Matter, and Surface Sediment of a Turbid River. Sci. Total Environ..

[52] Paige T., De Silva T., Buddhadasa S., Prasad S., Nugegoda D., Pettigrove V. (2024). Background Concentrations and Spatial Distribution of PFAS in Surface Waters and Sediments of the Greater Melbourne Area, Australia. Chemosphere.

[53] Xie L.-N., Wang X.-C., Dong X.-J., Su L.-Q., Zhu H.-J., Wang C., Zhang D.-P., Liu F.-Y., Hou S.-S., Dong B. (2021). Concentration, Spatial Distribution, and Health Risk Assessment of PFASs in Serum of Teenagers, Tap Water and Soil near a Chinese Fluorochemical Industrial Plant. Environ. Int..

[54] Liu Z., Lu Y., Song X., Jones K., Sweetman A.J., Johnson A.C., Zhang M., Lu X., Su C. (2019). Multiple Crop Bioaccumulation and Human Exposure of Perfluoroalkyl Substances around a Mega Fluorochemical Industrial Park, China: Implication for Planting Optimization and Food Safety. Environ. Int..

[55] Besis A., Botsaropoulou E., Samara C., Katsoyiannis A., Hanssen L., Huber S. (2019). Perfluoroalkyl Substances (PFASs) in Air-Conditioner Filter Dust of Indoor Microenvironments in Greece: Implications for Exposure. Ecotoxicol. Environ. Saf..

[56] Nyström-Kandola J., Ahrens L., Glynn A., Johanson G., Benskin J.P., Gyllenhammar I., Lignell S., Vogs C. (2023). Low Concentrations of Perfluoroalkyl Acids (PFAAs) in Municipal Drinking Water Associated with Serum PFAA Concentrations in Swedish Adolescents. Environ. Int..

[57] Chang N., Cohen-Hubal E., Turpin B. Indoor Residential Exposure to PFAS and Emissions to the Outdoor Environment: Results from IPA Campaign. Proceedings of the AAAR 42nd Annual Conference.

[58] Yao J., Dong Z., Jiang L., Pan Y., Zhao M., Bai X., Dai J. (2023). Emerging and Legacy Perfluoroalkyl Substances in Breastfed Chinese Infants: Renal Clearance, Body Burden, and Implications. Environ. Health Perspect..

[59] Pérez F., Nadal M., Navarro-Ortega A., Fàbrega F., Domingo J.L., Barceló D., Farré M. (2013). Accumulation of Perfluoroalkyl Substances in Human Tissues. Environ. Int..

[60] Nilsson H., Kärrman A., Rotander A., Van Bavel B., Lindström G., Westberg H. (2013). Professional Ski Waxers’ Exposure to PFAS and Aerosol Concentrations in Gas Phase and Different Particle Size Fractions. Environ. Sci. Process. Impacts.

[61] Poothong S., Thomsen C., Padilla-Sanchez J.A., Papadopoulou E., Haug L.S. (2017). Distribution of Novel and Well-Known Poly- and Perfluoroalkyl Substances (PFASs) in Human Serum, Plasma, and Whole Blood. Environ. Sci. Technol..

[62] Lucas K., Gaines L.G.T., Paris-Davila T., Nylander-French L.A. (2023). Occupational Exposure and Serum Levels of Per- and Polyfluoroalkyl Substances (PFAS): A Review. Am. J. Ind. Med..

[63] Ferber R., Noehren B., Hamill J., Davis I.S. (2010). Competitive Female Runners with a History of Iliotibial Band Syndrome Demonstrate Atypical Hip and Knee Kinematics. J. Orthop. Sports Phys. Ther..

[64] Zheng G., Eick S.M., Salamova A. (2023). Elevated Levels of Ultrashort- and Short-Chain Perfluoroalkyl Acids in US Homes and People. Environ. Sci. Technol..

[65] Xu Y., Fletcher T., Pineda D., Lindh C.H., Nilsson C., Glynn A., Vogs C., Norström K., Lilja K., Jakobsson K. (2020). Serum Half-Lives for Short- and Long-Chain Perfluoroalkyl Acids after Ceasing Exposure from Drinking Water Contaminated by Firefighting Foam. Environ. Health Perspect..

[66] Freberg B.I., Haug L.S., Olsen R., Daae H.L., Hersson M., Thomsen C., Thorud S., Becher G., Molander P., Ellingsen D.G. (2010). Occupational Exposure to Airborne Perfluorinated Compounds during Professional Ski Waxing. Environ. Sci. Technol..

[67] Calafat A.M., Kato K., Hubbard K., Jia T., Botelho J.C., Wong L.-Y. (2019). Legacy and Alternative Per- and Polyfluoroalkyl Substances in the U.S. General Population: Paired Serum-Urine Data from the 2013–2014 National Health and Nutrition Examination Survey. Environ. Int..

[68] Halldorsson T.I., Rytter D., Haug L.S., Bech B.H., Danielsen I., Becher G., Henriksen T.B., Olsen S.F. (2012). Prenatal Exposure to Perfluorooctanoate and Risk of Overweight at 20 Years of Age: A Prospective Cohort Study. Environ. Health Perspect..

[69] Mora A.M., Oken E., Rifas-Shiman S.L., Webster T.F., Gillman M.W., Calafat A.M., Ye X., Sagiv S.K. (2017). Prenatal Exposure to Perfluoroalkyl Substances and Adiposity in Early and Mid-Childhood. Environ. Health Perspect..

[70] Braun J.M., Chen A., Romano M.E., Calafat A.M., Webster G.M., Yolton K., Lanphear B.P. (2016). Prenatal Perfluoroalkyl Substance Exposure and Child Adiposity at 8 Years of Age: The HOME Study. Obesity.

[71] Lum K.J., Sundaram R., Barr D.B., Louis T.A., Buck Louis G.M. (2017). Perfluoroalkyl Chemicals, Menstrual Cycle Length, and Fecundity: Findings from a Prospective Pregnancy Study. Epidemiology.

[72] Zhang N., Wang W., Li W., Liu C., Chen Y., Yang Q., Wang Y., Sun K. (2015). Inhibition of 11β-HSD2 Expression by Triclosan via Induction of Apoptosis in Human Placental Syncytiotrophoblasts. J. Clin. Endocrinol. Metab..

[73] Karnes C., Winquist A., Steenland K. (2014). Incidence of Type II Diabetes in a Cohort with Substantial Exposure to Perfluorooctanoic Acid. Environ. Res..

[74] Henry B.J., Carlin J.P., Hammerschmidt J.A., Buck R.C., Buxton L.W., Fiedler H., Seed J., Hernandez O. (2018). A Critical Review of the Application of Polymer of Low Concern and Regulatory Criteria to Fluoropolymers. Integr. Environ. Assess. Manag..

[75] Schlummer M., Sölch C., Meisel T., Still M., Gruber L., Wolz G. (2015). Emission of Perfluoroalkyl Carboxylic Acids (PFCA) from Heated Surfaces Made of Polytetrafluoroethylene (PTFE) Applied in Food Contact Materials and Consumer Products. Chemosphere.

[76] Seacat A.M., Thomford P.J., Hansen K.J., Olsen G.W., Case M.T., Butenhoff J.L. (2002). Subchronic Toxicity Studies on Perfluorooctanesulfonate Potassium Salt in Cynomolgus Monkeys. Toxicol. Sci..

[77] Elcombe C.R., Elcombe B.M., Foster J.R., Chang S.-C., Ehresman D.J., Butenhoff J.L. (2012). Hepatocellular Hypertrophy and Cell Proliferation in Sprague-Dawley Rats from Dietary Exposure to Potassium Perfluorooctanesulfonate Results from Increased Expression of Xenosensor Nuclear Receptors PPARα and CAR/PXR. Toxicology.

[78] Qazi M.R., Hassan M., Nelson B.D., De Pierre J.W., Abedi-Valugerdi M. (2013). Both Sub-Acute, Moderate-Dose and Short-Term, Low-Dose Dietary Exposure of Mice to Perfluorooctane Sulfonate Exacerbates Concanavalin A-Induced Hepatitis. Toxicol. Lett..

[79] Amstutz V.H., Cengo A., Gehres F., Sijm D.T.H.M., Vrolijk M.F. (2022). Investigating the Cytotoxicity of Per- and Polyfluoroalkyl Substances in HepG2 Cells: A Structure-Activity Relationship Approach. Toxicology.

[80] Das K.P., Wood C.R., Lin M.T., Starkov A.A., Lau C., Wallace K.B., Corton J.C., Abbott B.D. (2017). Perfluoroalkyl Acids-Induced Liver Steatosis: Effects on Genes Controlling Lipid Homeostasis. Toxicology.

[81] Chengelis C.P., Kirkpatrick J.B., Radovsky A., Shinohara M. (2009). A 90-Day Repeated Dose Oral (Gavage) Toxicity Study of Perfluorohexanoic Acid (PFHxA) in Rats (with Functional Observational Battery and Motor Activity Determinations). Reprod. Toxicol..

[82] Mahapatra C.T., Damayanti N.P., Guffey S.C., Serafin J.S., Irudayaraj J., Sepúlveda M.S. (2017). Comparative In Vitro Toxicity Assessment of Perfluorinated Carboxylic Acids. J. Appl. Toxicol..

[83] Liu H., Sun W., Zhou Y., Griffin N., Faulkner S., Wang L. (2022). iTRAQ-Based Quantitative Proteomics Analysis of Sprague-Dawley Rats Liver Reveals Perfluorooctanoic Acid-Induced Lipid Metabolism and Urea Cycle Dysfunction. Toxicol. Lett..

[84] Foreman J.E., Chang S.-C., Ehresman D.J., Butenhoff J.L., Anderson C.R., Palkar P.S., Kang B.-H., Gonzalez F.J., Peters J.M. (2009). Differential Hepatic Effects of Perfluorobutyrate Mediated by Mouse and Human PPAR-Alpha. Toxicol. Sci..

[85] Ikeda T., Aiba K., Fukuda K., Tanaka M. (1985). The Induction of Peroxisome Proliferation in Rat Liver by Perfluorinated Fatty Acids, Metabolically Inert Derivatives of Fatty Acids. J. Biochem..

[86] Li D., Zhang L., Zhang Y., Guan S., Gong X., Wang X. (2019). Maternal Exposure to Perfluorooctanoic Acid (PFOA) Causes Liver Toxicity through PPAR-α Pathway and Lowered Histone Acetylation in Female Offspring Mice. Environ. Sci. Pollut. Res. Int..

[87] Robarts D.R., Paine-Cabrera D., Kotulkar M., Venneman K.K., Gunewardena S., Foquet L., Bial G., Apte U. (2024). Identifying Novel Mechanisms of Per- and Polyfluoroalkyl Substance-Induced Hepatotoxicity Using FRG Humanized Mice. Arch. Toxicol..

[88] Wan H.T., Zhao Y.G., Wei X., Hui K.Y., Giesy J.P., Wong C.K.C. (2012). PFOS-Induced Hepatic Steatosis, the Mechanistic Actions on β-Oxidation and Lipid Transport. Biochim. Biophys. Acta.

[89] Attema B., Janssen A.W.F., Rijkers D., van Schothorst E.M., Hooiveld G.J.E.J., Kersten S. (2022). Exposure to Low-Dose Perfluorooctanoic Acid Promotes Hepatic Steatosis and Disrupts the Hepatic Transcriptome in Mice. Mol. Metab..

[90] Darbre P.D. (2017). Endocrine Disruptors and Obesity. Curr. Obes. Rep..

[91] Schlezinger J.J., Hyötyläinen T., Sinioja T., Boston C., Puckett H., Oliver J., Heiger-Bernays W., Webster T.F. (2021). Perfluorooctanoic Acid Induces Liver and Serum Dyslipidemia in Humanized PPARα Mice Fed an American Diet. Toxicol. Appl. Pharmacol..

[92] Stoffels C.B.A., Angerer T.B., Robert H., Poupin N., Lakhal L., Frache G., Mercier-Bonin M., Audinot J.-N. (2023). Lipidomic Profiling of PFOA-Exposed Mouse Liver by Multi-Modal Mass Spectrometry Analysis. Anal. Chem..

[93] Schlezinger J.J., Puckett H., Oliver J., Nielsen G., Heiger-Bernays W., Webster T.F. (2020). Perfluorooctanoic Acid Activates Multiple Nuclear Receptor Pathways and Skews Expression of Genes Regulating Cholesterol Homeostasis in Liver of Humanized PPARα Mice Fed an American Diet. Toxicol. Appl. Pharmacol..

[94] Jiang L., Hong Y., Xie G., Zhang J., Zhang H., Cai Z. (2021). Comprehensive Multi-Omics Approaches Reveal the Hepatotoxic Mechanism of Perfluorohexanoic Acid (PFHxA) in Mice. Sci. Total Environ..

[95] Abdellatif A., Al-Tonsy A.H., Awad M.E., Roberfroid M., Khan M.N.U. (2003). Peroxisomal Enzymes and 8-Hydroxydeoxyguanosine in Rat Liver Treated with Perfluorooctanoic Acid. Dis. Markers.

[96] Trosko J.E., Upham B.L. (2005). The Emperor Wears No Clothes in the Field of Carcinogen Risk Assessment: Ignored Concepts in Cancer Risk Assessment. Mutagenesis.

[97] Upham B.L., Park J.-S., Babica P., Sovadinova I., Rummel A.M., Trosko J.E., Hirose A., Hasegawa R., Kanno J., Sai K. (2009). Structure-Activity-Dependent Regulation of Cell Communication by Perfluorinated Fatty Acids Using In Vivo and In Vitro Model Systems. Environ. Health Perspect..

[98] Meng X., Li S., Li Y., Gan R.-Y., Li H.-B. (2018). Gut Microbiota’s Relationship with Liver Disease and Role in Hepatoprotection by Dietary Natural Products and Probiotics. Nutrients.

[99] Choi J., Kim J.-Y., Lee H.-J. (2022). Human Evidence of Perfluorooctanoic Acid (PFOA) Exposure on Hepatic Disease: A Systematic Review and Meta-Analysis. Int. J. Environ. Res. Public Health.

[100] Sen P., Qadri S., Luukkonen P.K., Ragnarsdottir O., McGlinchey A., Jäntti S., Juuti A., Arola J., Schlezinger J.J., Webster T.F. (2022). Exposure to Environmental Contaminants Is Associated with Altered Hepatic Lipid Metabolism in Non-Alcoholic Fatty Liver Disease. J. Hepatol..

[101] Jain R.B. (2019). Concentration of Selected Liver Enzymes across the Stages of Glomerular Function: The Associations with PFOA and PFOS. Heliyon.

[102] Jin R., McConnell R., Catherine C., Xu S., Walker D.I., Stratakis N., Jones D.P., Miller G.W., Peng C., Conti D.V. (2020). Perfluoroalkyl Substances and Severity of Nonalcoholic Fatty Liver in Children: An Untargeted Metabolomics Approach. Environ. Int..

[103] Stratakis N., VConti D., Jin R., Margetaki K., Valvi D., Siskos A.P., Maitre L., Garcia E., Varo N., Zhao Y. (2020). Prenatal Exposure to Perfluoroalkyl Substances Associated with Increased Susceptibility to Liver Injury in Children. Hepatology.

[104] Lu Y., Gao K., Li X., Tang Z., Xiang L., Zhao H., Fu J., Wang L., Zhu N., Cai Z. (2019). Mass Spectrometry-Based Metabolomics Reveals Occupational Exposure to Per- and Polyfluoroalkyl Substances Relates to Oxidative Stress, Fatty Acid β-Oxidation Disorder, and Kidney Injury in a Manufactory in China. Environ. Sci. Technol..

[105] World Health Organization (2002). Global Assessment of the State-of-the-Science of Endocrine Disruptors.

[106] Borghoff S.J., Fitch S., Rager J.E., Huggett D. (2018). A Hypothesis-Driven Weight-of-Evidence Analysis to Evaluate Potential Endocrine Activity of Perfluorohexanoic Acid. Regul. Toxicol. Pharmacol..

[107] Razvi S., Jabbar A., Pingitore A., Danzi S., Biondi B., Klein I., Peeters R., Zaman A., Iervasi G. (2018). Thyroid Hormones and Cardiovascular Function and Diseases. J. AM. Coll. Cardiol..

[108] Chemical Evaluation and Research Institute (CERI) (2015). Fish Short Term Reproduction Assay of Perfluorohexanoic Acid, Ammonium Salt or Sodium Salt in Medaka.

[109] Wasel O., Thompson K.M., Gao Y., Godfrey A.E., Gao J., Mahapatra C.T., Lee L.S., Sepúlveda M.S., Freeman J.L. (2021). Comparison of Zebrafish In Vitro and In Vivo Developmental Toxicity Assessments of Perfluoroalkyl Acids (PFAAs). J. Toxicol. Environ. Health A.

[110] Kranthi Kumar K., Uma Devi B., Neeraja P. (2017). Integration of in Silico Approaches to Determination of Endocrine-Disrupting Perfluorinated Chemicals Binding Potency with Steroidogenic Acute Regulatory Protein. Biochem. Biophys. Res. Commun..

[111] Qiu Z., Qu K., Luan F., Liu Y., Zhu Y., Yuan Y., Li H., Zhang H., Hai Y., Zhao C. (2020). Binding Specificities of Estrogen Receptor with Perfluorinated Compounds: A Cross Species Comparison. Environ. Int..

[112] Zhang H., Lu Y., Luo B., Yan S., Guo X., Dai J. (2014). Proteomic Analysis of Mouse Testis Reveals Perfluorooctanoic Acid-Induced Reproductive Dysfunction via Direct Disturbance of Testicular Steroidogenic Machinery. J. Proteome Res..

[113] White S.S., Stanko J.P., Kato K., Calafat A.M., Hines E.P., Fenton S.E. (2011). Gestational and Chronic Low-Dose PFOA Exposures and Mammary Gland Growth and Differentiation in Three Generations of CD-1 Mice. Environ. Health Perspect..

[114] Zhao Y., Tan Y.S., Strynar M.J., Perez G., Haslam S.Z., Yang C. (2012). Perfluorooctanoic Acid Effects on Ovaries Mediate Its Inhibition of Peripubertal Mammary Gland Development in Balb/c and C57Bl/6 Mice. Reprod. Toxicol..

[115] Jain R.B. (2013). Association between Thyroid Profile and Perfluoroalkyl Acids: Data from NHNAES 2007-2008. Environ. Res..

[116] Melzer D., Rice N., Depledge M.H., Henley W.E., Galloway T.S. (2010). Association between Serum Perfluorooctanoic Acid (PFOA) and Thyroid Disease in the U.S. National Health and Nutrition Examination Survey. Environ. Health Perspect..

[117] Tan K., Zhang Q., Wang Y., Wang C., Hu C., Wang L., Liu H., Tian Z. (2024). Associations between Per- and Polyfluoroalkyl Substances Exposure and Thyroid Hormone Levels in the Elderly. Sci. Total Environ..

[118] Kim D.-H., Kim U.-J., Kim H.-Y., Choi S.-D., Oh J.-E. (2016). Perfluoroalkyl Substances in Serum from South Korean Infants with Congenital Hypothyroidism and Healthy Infants--Its Relationship with Thyroid Hormones. Environ. Res..

[119] Liu H., Pan Y., Jin S., Li Y., Zhao L., Sun X., Cui Q., Zhang B., Zheng T., Xia W. (2020). Associations of Per-/Polyfluoroalkyl Substances with Glucocorticoids and Progestogens in Newborns. Environ. Int..

[120] Liu H., Pan Y., Jin S., Sun X., Jiang Y., Wang Y., Ghassabian A., Li Y., Xia W., Cui Q. (2021). Associations between Six Common Per- and Polyfluoroalkyl Substances and Estrogens in Neonates of China. J. Hazard. Mater..

[121] Ballesteros V., Costa O., Iñiguez C., Fletcher T., Ballester F., Lopez-Espinosa M.-J. (2017). Exposure to Perfluoroalkyl Substances and Thyroid Function in Pregnant Women and Children: A Systematic Review of Epidemiologic Studies. Environ. Int..

[122] Kaczmarska I., Mokra K., Michałowicz J. (2025). Perfluorooctanoic Acid and Its Short-Chain Substitutes Induce Cytotoxic and Prooxidative Changes in Human Peripheral Blood Mononuclear Cells: A Comparative Study. Int. J. Mol. Sci..

[123] Agency for Toxic Substances and Disease Registry (2021). Toxicological Profile for Perfluoroalkyls.

[124] De Guise S., Levin M. (2021). Suppression of Th2 Cytokines as a Potential Mechanism for Reduced Antibody Response Following PFOA Exposure in Female B6C3F1 Mice. Toxicol. Lett..

[125] Dewitt J.C., Copeland C.B., Strynar M.J., Luebke R.W. (2008). Perfluorooctanoic Acid-Induced Immunomodulation in Adult C57BL/6J or C57BL/6N Female Mice. Environ. Health Perspect..

[126] Loveless S.E., Hoban D., Sykes G., Frame S.R., Everds N.E. (2008). Evaluation of the Immune System in Rats and Mice Administered Linear Ammonium Perfluorooctanoate. Toxicol. Sci..

[127] Iwai H., Yamashita K. (2006). A Fourteen-Day Repeated Dose Oral Toxicity Study of APFO in Rats. Drug Chem. Toxicol..

[128] Zhou Y., Hu L.-W., Qian Z., Geiger S.D., Parrish K.L., Dharmage S.C., Campbell B., Roponen M., Jalava P., Hirvonen M.-R. (2017). Interaction Effects of Polyfluoroalkyl Substances and Sex Steroid Hormones on Asthma among Children. Sci. Rep..

[129] Anderson-Mahoney P., Kotlerman J., Takhar H., Gray D., Dahlgren J. (2008). Self-Reported Health Effects among Community Residents Exposed to Perfluorooctanoate. New Solut..

[130] Dong G.-H., Tung K.-Y., Tsai C.-H., Liu M.-M., Wang D., Liu W., Jin Y.-H., Hsieh W.-S., Lee Y.L., Chen P.-C. (2013). Serum Polyfluoroalkyl Concentrations, Asthma Outcomes, and Immunological Markers in a Case-Control Study of Taiwanese Children. Environ. Health Perspect..

[131] Chang E.T., Adami H.-O., Boffetta P., Wedner H.J., Mandel J.S. (2016). A Critical Review of Perfluorooctanoate and Perfluorooctanesulfonate Exposure and Immunological Health Conditions in Humans. Crit. Rev. Toxicol..

[132] Lopez-Espinosa M.-J., Carrizosa C., Luster M.I., Margolick J.B., Costa O., Leonardi G.S., Fletcher T. (2021). Perfluoroalkyl Substances and Immune Cell Counts in Adults from the Mid-Ohio Valley (USA). Environ. Int..

[133] Innes K.E., Ducatman A.M., Luster M.I., Shankar A. (2011). Association of Osteoarthritis with Serum Levels of the Environmental Contaminants Perfluorooctanoate and Perfluorooctane Sulfonate in a Large Appalachian Population. Am. J. Epidemiol..

[134] Steenland K., Zhao L., Winquist A. (2015). A Cohort Incidence Study of Workers Exposed to Perfluorooctanoic Acid (PFOA). Occup. Environ. Med..

[135] Granum B., Haug L.S., Namork E., Stølevik S.B., Thomsen C., Aaberge I.S., van Loveren H., Løvik M., Nygaard U.C. (2013). Pre-Natal Exposure to Perfluoroalkyl Substances May Be Associated with Altered Vaccine Antibody Levels and Immune-Related Health Outcomes in Early Childhood. J. Immunotoxicol..

[136] Knudsen A.-K.S., Long M., Pedersen H.S., Bonefeld-Jørgensen E.C. (2018). Persistent Organic Pollutants and Haematological Markers in Greenlandic Pregnant Women: The ACCEPT Sub-Study. Int. J. Circumpolar Health.

[137] Impinen A., Longnecker M.P., Nygaard U.C., London S.J., Ferguson K.K., Haug L.S., Granum B. (2019). Maternal Levels of Perfluoroalkyl Substances (PFASs) during Pregnancy and Childhood Allergy and Asthma Related Outcomes and Infections in the Norwegian Mother and Child (MoBa) Cohort. Environ. Int..

[138] Goudarzi H., Miyashita C., Okada E., Kashino I., Kobayashi S., Chen C.-J., Ito S., Araki A., Matsuura H., Ito Y.M. (2016). Effects of Prenatal Exposure to Perfluoroalkyl Acids on Prevalence Ofallergic Diseases among 4-Year-Old Children. Environ. Int..

[139] Abraham K., El-Khatib A.H., Schwerdtle T., Monien B.H. (2021). Perfluorobutanoic Acid (PFBA): No High-Level Accumulation in Human Lung and Kidney Tissue. Int. J. Hyg. Environ. Health.

[140] Mogensen U.B., Grandjean P., Heilmann C., Nielsen F., Weihe P., Budtz-Jørgensen E. (2015). Structural Equation Modeling of Immunotoxicity Associated with Exposure to Perfluorinated Alkylates. Environ. Health.

[141] Looker C., Luster M.I., Calafat A.M., Johnson V.J., Burleson G.R., Burleson F.G., Fletcher T. (2014). Influenza Vaccine Response in Adults Exposed to Perfluorooctanoate and Perfluorooctanesulfonate. Toxicol. Sci..

[142] Grandjean P., Timmermann C.A.G., Kruse M., Nielsen F., Vinholt P.J., Boding L., Heilmann C., Mølbak K. (2020). Severity of COVID-19 at Elevated Exposure to Perfluorinated Alkylates. PLoS ONE.

[143] Farsetti A., Illi B., Gaetano C. (2023). How Epigenetics Impacts on Human Diseases. Eur. J. Intern. Med..

[144] Liu W., Irudayaraj J. (2020). Perfluorooctanoic Acid (PFOA) Exposure Inhibits DNA Methyltransferase Activities and Alters Constitutive Heterochromatin Organization. Food Chem. Toxicol..

[145] Ahmad S., Wen Y., Irudayaraj J.M.K. (2021). PFOA Induces Alteration in DNA Methylation Regulators and SARS-CoV-2 Targets Ace2 and Tmprss2 in Mouse Lung Tissues. Toxicol. Rep..

[146] Wen Y., Chen J., Li J., Arif W., Kalsotra A., Irudayaraj J. (2020). Effect of PFOA on DNA Methylation and Alternative Splicing in Mouse Liver. Toxicol. Lett..

[147] O’Brien M.L., Spear B.T., Glauert H.P. (2005). Role of Oxidative Stress in Peroxisome Proliferator-Mediated Carcinogenesis. Crit. Rev. Toxicol..

[148] Temkin A.M., Hocevar B.A., Andrews D.Q., Naidenko O.V., Kamendulis L.M. (2020). Application of the Key Characteristics of Carcinogens to Per and Polyfluoroalkyl Substances. Int. J. Environ. Res. Public Health.

[149] Eriksen K.T., Raaschou-Nielsen O., Sørensen M., Roursgaard M., Loft S., Møller P. (2010). Genotoxic Potential of the Perfluorinated Chemicals PFOA, PFOS, PFBS, PFNA and PFHxA in Human HepG2 Cells. Mutat. Res..

[150] Crebelli R., Caiola S., Conti L., Cordelli E., De Luca G., Dellatte E., Eleuteri P., Iacovella N., Leopardi P., Marcon F. (2019). Can Sustained Exposure to PFAS Trigger a Genotoxic Response? A Comprehensive Genotoxicity Assessment in Mice after Subacute Oral Administration of PFOA and PFBA. Regul. Toxicol. Pharmacol..

[151] Li L.-Y., Guan Y., Chen X.-S., Yang J.-M., Cheng Y. (2020). DNA Repair Pathways in Cancer Therapy and Resistance. Front. Pharmacol..

[152] Qu Y., Sheng N., Ji S., Li Z., Wang J., Pan Y., Hu X., Zheng X., Li Y., Song H. (2024). Dietary Seafood as a Potential Modifier in the Relationship between Per- and Polyfluoroalkyl Substances (PFASs) Burden and Prediabetes/Diabetes: Insights from a Nationally Representative Cross-Sectional Study. J. Hazard. Mater..

[153] (2025). IARC Working Group on the Identification of Carcinogenic Hazards to Humans. Perfluorooctanoic Acid (PFOA) and Perfluorooctanesulfonic Acid (PFOS).

[154] Pierozan P., Jerneren F., Karlsson O. (2018). Perfluorooctanoic Acid (PFOA) Exposure Promotes Proliferation, Migration and Invasion Potential in Human Breast Epithelial Cells. Arch. Toxicol..

[155] Bartell S.M., Vieira V.M. (2021). Critical Review on PFOA, Kidney Cancer, and Testicular Cancer. J. Air Waste Manag. Assoc..

[156] Barry V., Winquist A., Steenland K. (2013). Perfluorooctanoic Acid (PFOA) Exposures and Incident Cancers among Adults Living near a Chemical Plant. Environ. Health Perspect..

[157] Girardi P., Merler E. (2019). A Mortality Study on Male Subjects Exposed to Polyfluoroalkyl Acids with High Internal Dose of Perfluorooctanoic Acid. Environ. Res..

[158] Shearer J.J., Callahan C.L., Calafat A.M., Huang W.-Y., Jones R.R., Sabbisetti V.S., Freedman N.D., Sampson J.N., Silverman D.T., Purdue M.P. (2021). Serum Concentrations of Per- and Polyfluoroalkyl Substances and Risk of Renal Cell Carcinoma. J. Natl. Cancer Inst..

[159] Chang V.C., Rhee J., Berndt S.I., Moore S.C., Freedman N.D., Jones R.R., Silverman D.T., Gierach G.L., Hofmann J.N., Purdue M.P. (2023). Serum Perfluorooctane Sulfonate and Perfluorooctanoate and Risk of Postmenopausal Breast Cancer According to Hormone Receptor Status: An Analysis in the Prostate, Lung, Colorectal and Ovarian Cancer Screening Trial. Int. J. Cancer.

[160] Zahm S., Bonde J.P., Chiu W.A., Hoppin J., Kanno J., Abdallah M., Blystone C.R., Calkins M.M., Dong G.-H., Dorman D.C. (2024). Carcinogenicity of Perfluorooctanoic Acid and Perfluorooctanesulfonic Acid. Lancet Oncol..

[161] Davis J.A., Taylor M.M., Kraft A., Lambert J.C., Radke E., Schlosser P., Angrish M., Arzuaga X., Congleton J., Druwe I. (2022). IRIS Toxicological Review of Perfluorobutanoic Acid (PFBA, CASRN 375-22-4) and Related Salts.

[162] Klaunig J.E., Shinohara M., Iwai H., Chengelis C.P., Kirkpatrick J.B., Wang Z., Bruner R.H. (2015). Evaluation of the Chronic Toxicity and Carcinogenicity of Perfluorohexanoic Acid (PFHxA) in Sprague-Dawley Rats. Toxicol. Pathol..

[163] Janssen A.W.F., Jansen Holleboom W., Rijkers D., Louisse J., Hoekstra S.A., Schild S., Vrolijk M.F., Hoogenboom R.L.A.P., Beekmann K. (2024). Determination of In Vitro Immunotoxic Potencies of a Series of Perfluoralkylsubstances (PFASs) in Human Namalwa B Lymphocyte and Human Jurkat T Lymphocyte Cells. Front. Toxicol..

[164] Di Nisio A., Rocca M.S., De Toni L., Sabovic I., Guidolin D., Dall’Acqua S., Acquasaliente L., De Filippis V., Plebani M., Foresta C. (2020). Endocrine Disruption of Vitamin D Activity by Perfluoro-Octanoic Acid (PFOA). Sci. Rep..

[165] Kabakci R., Clark K.L., Plewes M.R., Monaco C.F., Davis J.S. (2023). Perfluorooctanoic Acid (PFOA) Inhibits Steroidogenesis and Mitochondrial Function in Bovine Granulosa Cells in Vitro. Environ. Pollut..

[166] Rashid F., Ahmad S., Irudayaraj J.M.K. (2020). Effect of Perfluorooctanoic Acid on the Epigenetic and Tight Junction Genes of the Mouse Intestine. Toxics.

